# Suspension of oral hygiene practices highlights key bacterial shifts in saliva, tongue, and tooth plaque during gingival inflammation and resolution

**DOI:** 10.1038/s43705-023-00229-5

**Published:** 2023-03-25

**Authors:** Michael William Hall, Nimali Chandhema Wellappuli, Ruo Chen Huang, Kay Wu, David King Lam, Michael Glogauer, Robert Gerald Beiko, Dilani Braziunas Senadheera

**Affiliations:** 1 Faculty of Graduate Studies, Dalhousie University, Halifax, NS Canada; 2 Faculty of Dentistry, University of Toronto, Toronto, ON Canada; 3 McMaster University, Hamilton, ON Canada; 4 University of the Pacific, San Francisco, CA USA; 5 Faculty of Computer Science, Dalhousie University, Halifax, NS Canada; 6 Stony Brook University, School of Dental Medicine, Stony Brook, NY USA

**Keywords:** Microbiome, Biomarkers, Population dynamics

## Abstract

Experimentally induced gingivitis is associated with inflammatory and microbiological changes in an otherwise healthy subject, demonstrating the impacts of discontinuing oral hygiene routines. Understanding the bacterial dynamics during the induction and resolution of gingival inflammation will aid in the development of bacterial prognostic tests and probiotics for severe oral disease. We profiled the bacterial community in 15 healthy subjects who suspended all oral-hygiene practices for three weeks. Saliva, tongue, subgingival, and supragingival plaque samples were collected over seven weeks and showed a return to community baseline after oral hygiene practices were resumed. Stronger temporal changes in subgingival and supragingival plaque suggest these sample types may be preferred over saliva or tongue plaque for future prognostics. Taxonomic groups spanning ten phyla demonstrated consistent abundance shifts, including a significant decrease in *Streptococcus*, *Neisseria*, and *Actinomyces* populations, and an increase in *Prevotella*, *Fusobacterium*, and *Porphyromonas* populations. With four distinct oral sites surveyed and results mapped to the Human Oral Microbiome Database reference set, this work provides a comprehensive taxonomic catalog of the bacterial shifts observed during the onset and resolution of gingival inflammation.

## Introduction

Periodontitis is an infectious, inflammatory disease that is characterized by the destruction of tooth-supporting structures that include the alveolar bone, gingiva, and connective tissue [1–3]. The mildest form of periodontal disease that precedes periodontitis is known as gingivitis [4]. It is characterized by gingival inflammation in response to dental plaque accumulation, which can be restored to health with proper oral hygiene that reduces the bacterial burden. Left untreated, gingivitis can proceed to chronic periodontitis causing irreversible bone and tissue damage. Gingivitis, the early and reversible stage of the disease, therefore represents a critical window to prevent further progression of disease and preclude permanent loss of bone and tissue. Hence, an understanding of the dynamic composition of the oral microbiota during the early stages of transition from health to disease can help identify useful prognostic targets and novel treatment modalities to halt or reverse disease progression before permanent damage occurs.

The pathogenesis of periodontitis is multifactorial, and involves complex interactions between host microbiota, immunity, and environmental factors such as smoking and diet [1]. The causative role of plaque in the development of periodontal disease was first demonstrated using an experimental gingivitis model (EGM), by Löe et al. in 1965 [5]. The non-invasive EGM is comprised of two phases that allow the controlled induction and resolution of gingivitis in subjects with healthy gingiva. The induction phase is initiated by temporarily discontinuing all forms of oral hygiene practices (OHP) over several weeks. In the restoration phase that follows, gingival health is returned to baseline by resuming OHP, thus precluding permanent damage to the bones and dentition of study participants [5]. EGM facilitates a non-invasive model in humans for longitudinal surveillance of oral microbiota and other parameters with disease progression [5–11].

Cumulative evidence from 16S rRNA gene and shotgun metagenomic sequencing studies supports the existence of distinct bacterial community structures in health and periodontal disease [12–18]. A comparably smaller number of human studies have been conducted focusing on temporal changes in the oral microbiome with early gingivitis onset, as opposed to more severe and progressed periodontal disease e.g., [6–10, 19, 20]. Further, most studies examining the early stages of disease have examined only the bacterial burden in a single location (e.g., subgingivae; [7, 9, 10, 20]) or with a limited number of time points (three or fewer) [5, 7, 8]. However, the oral cavity contains heterogeneous niches such as tissues of the tongue, saliva, and hard tooth surfaces which harbor distinct microbiologically complex communities [21–24], which are yet to be characterized under longitudinal disease progression.

In this article, we present the results of an EGM study that comprehensively characterizes how hundreds of bacterial taxa identified in saliva, supragingival and subgingival plaque, and tongue dorsum sites change in community composition on a weekly basis over three weeks in 15 individuals when oral hygiene procedures are stopped. Resuming oral hygiene in the last two weeks of the study restored gum health, confirmed in the original Wellappuli et al. [25] study by a decrease in bleeding and return to baseline inflammatory marker levels. In this work, we observed a commensurate restoration of many bacterial groups that were reduced in the transition from healthy to diseased states, reinforcing results from a recent EGM study that indicated a return to the healthy baseline microbial community [10].

## Subjects and methods

### Participant demographics and sample collection

Samples were originally collected for a single-arm clinical trial that was designated to characterize oral and circulatory polymorphonuclear neutrophils during the onset and resolution of gingivitis [25]. Subjects were recruited through the University of Toronto, Faculty of Dentistry as per guidelines of the Research Ethics Board (REB 30044). All had given informed consent to participate and followed the complete trial design. Oral health status of participants was determined by a dentist who performed a full-mouth clinical examination that included the inspection of teeth, oral mucosa and periodontal status. All subjects were systemically healthy, with no active or previous history of periodontal disease, chronic aphthous ulcers or tonsillitis, and no more than four active or filled cavitated lesions. The absence of periodontitis was clinically assessed on all patients through analysis of tissue inflammation, bleeding on probing scores, and absence of periodontal destruction including clinical attachment loss. None were pregnant, past or current smokers, or users of non-steroidal anti-inflammatory or anti-microbial drugs, mouthwashes, or vitamin supplements within the previous three months. A subset of fifteen of the original Wellappuli et al. [25] study’s subjects (seven males and eight females aged 19–29 years) were selected to have their samples taxonomically profiled for the present work.

**Fig. 1:**
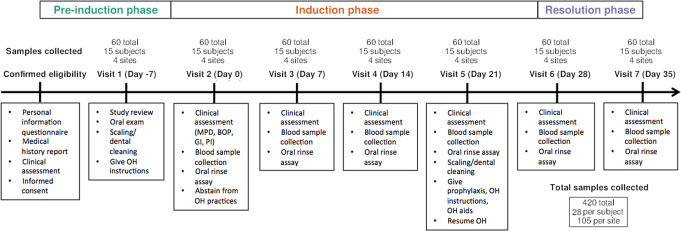
Study timeline and measurements at each visit. OH oral hygiene; MPD mean pocket depth; BOP bleeding on probing; GI gingival index; PI plaque index.

Each individual had seven study visits (Fig. 1). All plaque samples were collected at the beginning of each visit, before any interventions or assessments were conducted. Only one experienced and trained clinician was employed to collect all samples, thereby maximizing consistency and minimizing inter-individual discrepancy. The pre-trial preparation took place 7 days prior (day −7) to baseline (day 0), where an oral exam, scaling, and cleaning were performed and oral hygiene instructions were given for the next 7 days. At day 0, subjects were asked to abstain from all oral hygiene practices, including chewing gum, for a period of 21 days. During the induction phase (day 0, day 7, day 14, and day 21) clinical assessment involving full mouth pocket depth measurements and bleeding on probing were performed on all teeth except the third molars as they were not present in all subjects. The clinical examinations were done using a University of North Carolina Probe. Gingival index (GI) [26] and plaque index (PI) [26, 27] were recorded for the six surfaces of each tooth (disto-buccal, middle, mesio-buccal, mesio-lingual/mesio-palatal, middle, disto-lingual/disto-buccal). Oral rinse and blood samples were also collected to be used for another study investigating the relationship between inflammatory host response and gingivitis. At the end of day 21, subjects received professional prophylaxis, and were instructed to resume normal hygiene practices. They received fresh toothbrushes to facilitate the resolution of gingival inflammation over the recovery phase to avoid microbial input from previously used toothbrushes. For another 2 weeks—at day 28 and day 35—subjects underwent final clinical assessments and sample collection to confirm restoration of gingival health.

Collection of all oral samples was performed by the same clinician. Subgingival, supragingival, and tongue samples were collected by using separate dental curettes. Subgingival and supragingival plaques were obtained from Ramford’s 6 teeth [28–32]. Plaque from tongue was collected by carrying out midline scrapings for a maximum of four times along the dorsum of the tongue using sterile 10 µl plastic inoculation loop. Stimulated salivary samples were collected in conical tubes and submerged on ice. Participants were asked to chew on a piece of plastic parafilm for 30 s and subsequently swallowed the first saliva. Afterwards, participants expectorated once every 30 s and a total of 4–5 ml of saliva was collected each time into the conical tube. Salivary flow rate was calculated by dividing the total volume of saliva accumulated by the time required for collection. Saliva was aliquoted into sterile 1.5 ml microcentrifuge tubes (Eppendorf; Axygen, CA, USA) for storage at −80 °C until further processing.

### DNA extraction, sequencing, and quantification

Amplification and sequencing of the 16S rRNA gene was performed at the Integrated Microbiome Resource at Dalhousie University as described in Comeau et al. [33] and Walters et al. [34]. DNA was extracted from 500 µl of plaque using the DNeasy PowerSoil Kit (QIAGEN) according to the manufacturer’s instructions, with the exception of several modifications. The plaque samples (all except saliva) were homogenized by 15 passages through a 3 ml syringe with an 18 G × 1.5 in. needle prior to extraction. The DNeasy PowerSoil Kit’s PowerBead Tubes were vortexed using Thermo Savant FastPrep FP120 Cell Homogenizer at 5 m/s for 45 s. Samples were divided into two tubes prior to adding Solution C3 of the DNeasy PowerSoil Kit and subsequently mixed into a single spin filter. Final elution of DNA was completed with 150 µl of Solution C6 of the DNeasy PowerSoil Kit. DNA concentrations were determined by UV spectrophotometry (Ultrospec 3000, Pharmacia Biotech) at a wavelength of 260 nm.

A one-step PCR approach is used to attach dual indexes, adaptors, and primers, followed by PCR amplification in duplicate with 35 cycles, pooling, and clean up and normalization with the Invitrogen SequalPrep kit. Primers used were 515FB (5′-GTGYCAGCMGCCGCGGTAA-3′) and 926 R (5′-CCGYCAATTYMTTTRAGTTT-3′) which span the V4-V5 hypervariable regions. PCR amplicons were purified and sequenced on an Illumina MiSeq to produce 300 bp paired-end reads.

### Computational analysis

Sequences were processed using the QIIME2 software library (version 2022.2) [35]. Sequence filtering, denoising, merging, and amplicon sequence variant (ASV) clustering was completed using DADA2 (version 1.22.0) via the q2-dada2 plugin [36]. Primer sequences were trimmed from forward and reverse reads before paired-end assembly. Taxonomic classification labels were assigned with the q2-feature-classifier using the full-length, 99% pre-clustered SILVA 138 pre-trained Naïve Bayes classifier artifact [37]. Alpha diversity analyses were performed with the q2-diversity plugin.

To account for the compositional nature of the data, the ASV table and collapsed taxonomic tables were transformed using a centered log ratio (CLR) transform before analyzing for trends using the compositions R library (version 2.0–4) [38]. This allowed for trends during the induction phase to be modeled using a generalized linear model via the lme4 toolkit (version 1.1–29) [39]. The model used is described by the lme4 formulavalue ∼ time + (1|subject) describing a repeated measures experiment, which fits a slope to the transformed relative abundances as a function of time. The value parameter represents the CLR transformed abundance, diversity measure, or periodontal health measure (bleeding score etc.). Significant slopes were assessed with the anova.lmerModLmerTest function from the lmerTest package (version 3.1–3) [40], which uses Satterthwaite’s method to estimate degrees of freedom and F-statistics. Multiple hypothesis test correction was completed using false discovery rate correction for microbial abundance tests.

Sequence variants that demonstrated a significant positive or negative slope during the induction period were aligned alongside the extended Human Oral Microbiome Database (eHOMD) reference sequences (version 15.22) after being trimmed to the same region sequenced. Combined significant ASV and eHOMD sequence alignments were generated using MAFFT (version 7.490) and trees were generated using FastTree (version 2.1.10) via the q2-phylogeny plugin [41, 42]. Phylogenetic trees were visualized using ggtree (version 3.2.1) [43].

## Results

### High-throughput sequencing of 16S rRNA marker genes

Post filtering and processing of 420 plaque and saliva samples, 12,899,647 16S rRNA gene sequences were obtained and clustered into 11,145 ASVs. Samples had a minimum of 4222, maximum of 78,879, and median of 29,220 sequences. Rarefaction plots computed with the Shannon, Faith’s phylogenetic diversity, and observed ASV measures demonstrated a plateau in taxon accumulation (data not shown), suggesting the depth of sequencing was sufficient to capture the majority of the abundant taxa.

### Quantifying bacterial diversity

**Fig. 2:**
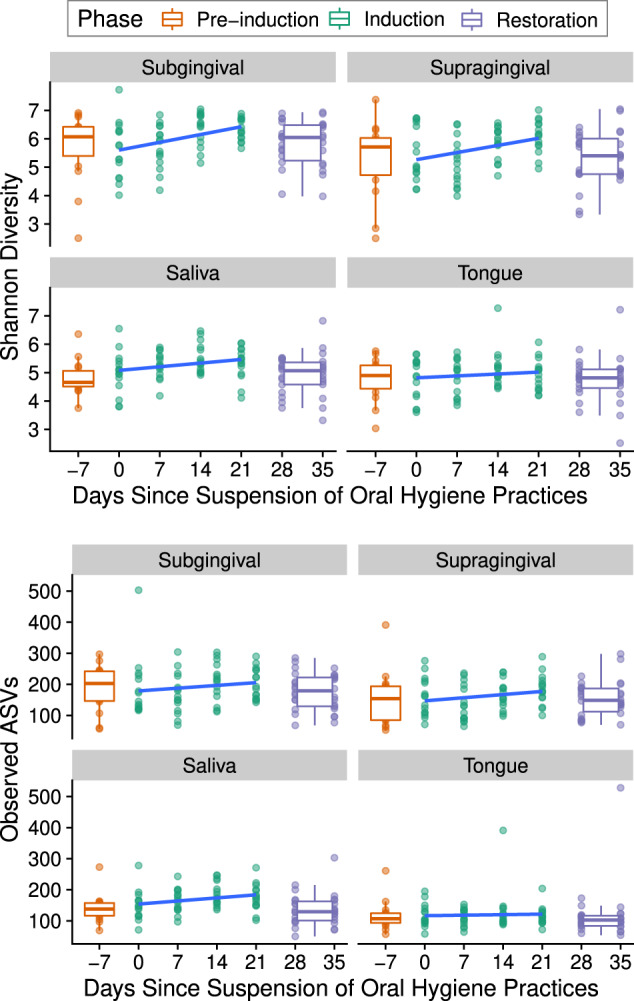
Alpha diversity over time in experimental gingivitis. Shannon diversity and Observed ASV measures shown for each of the 420 samples. Boxplots show the median, 1st, and 3rd quartiles, and 1.5 times the inter-quartile range. Slope over induction phase (computed by GLM) shown in blue. No slopes were found to be statistically significant over the induction phase (*p* > 0.05).

The alpha diversity was measured using the Shannon diversity and Observed ASV measures and increased on average during the gingivitis induction phase in all four oral sites sampled (Fig. 2). The observed increase in alpha diversity was not statistically significant over the induction phase, as determined by repeated measure generalized linear model testing (*p* < 0.05 for all alpha diversity measures and sites). The magnitude of the slopes was largest on average in the subgingival site, followed by the supragingival site, saliva, and finally the tongue dorsum (Fig. 2).

Changes in inter-sample (beta) diversity were measured by Bray–Curtis dissimilarities. Within-subject dissimilarities were consistently lower than between-subject dissimilarities at any given timepoint (Fig. S21). In terms of inter-individual diversity, the tongue and saliva samples were generally more similar across individuals than the sub- and supragingival plaque samples. Mean (±standard deviation) inter-individual saliva and tongue dissimilarity scores were 0.608 ± 0.113 and 0.618 ± 0.131 while subgingival and supragingival scores were 0.738 ± 0.084 and 0.713 ± 0.097, respectively.

### Significant microbial abundance trends

**Fig. 3:**
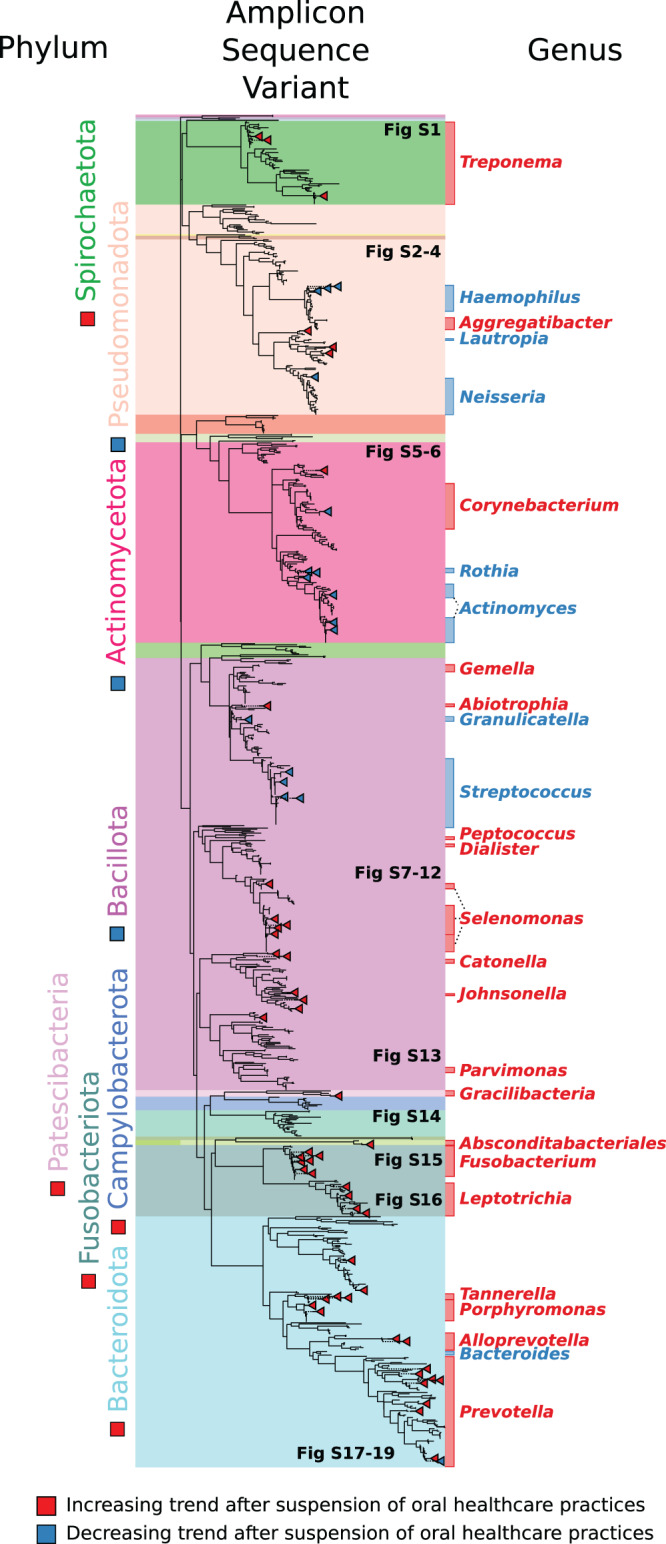
Summary of differentially abundant bacteria after suspension of oral health practices. A summary of the differentially abundant phyla, genera, and ASVs. Phylogenetic tree represents eHOMD V15.22 16S rRNA gene sequences trimmed to the V4-V5 region, with differential ASVs shown by red or blue circles. Regions of the tree are colored by phylum-level classifications of the reference sequences. Red squares, triangles, and genus labels indicate taxa that increased on aggregate over the gingivitis induction phase, with blue indicating a decreasing trend. Red and blue bars on the right side show the span of the significant genera on the tree.

Although aggregate quantifications of microbial diversity did not statistically increase over the induction phase, many taxonomic groups demonstrated significant abundance trends. All ASV representative sequences were taxonomically classified, with read counts aggregated at the phylum, genus, and species levels. Each taxonomic grouping had its CLR transformed read counts tested for a non-zero slope over the induction phase. After p-value adjustment for multiple hypothesis test correction, 10 phyla, 40 genus-level designations, 43 species-level designations, and 66 ASVs were found to be significantly increasing or decreasing in relative proportion over the gingivitis induction phase. A graphical summary of the results at the phylum, genus, and ASV level is shown in Fig. 3 with full results in File S1.

**Fig. 4:**
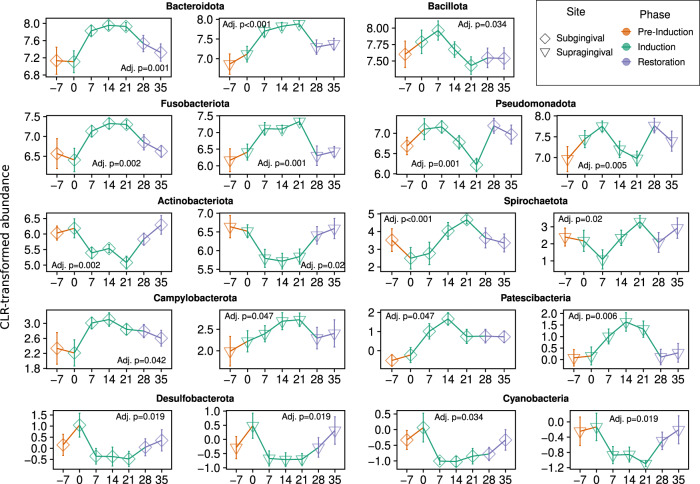
Differentially abundant phyla after the suspension of oral health practices. Phyla with abundances that demonstrated positive slopes over the gingivitis induction period. Sampling time points are on the *x*-axis and CLR-transformed abundance values are on the *y*-axis. *P* values are FDR-adjusted for multiple hypothesis test correction, with all results *p* ≤ 0.05 shown.

**Fig. 5:**
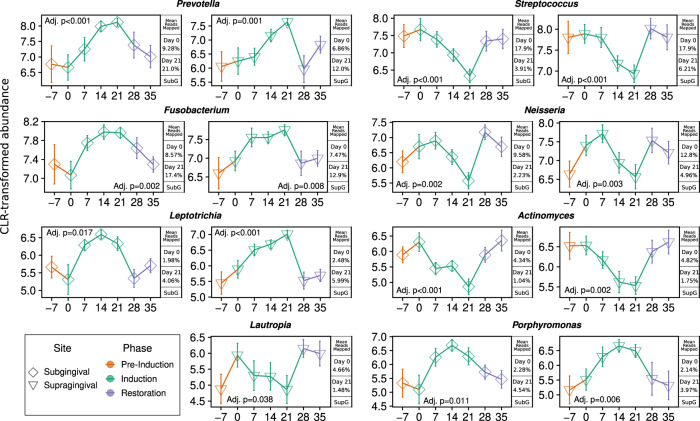
Differentially abundant genera after the suspension of oral health practices. Genera with abundances that demonstrated positive slopes over the gingivitis induction period. Top eight genera by relative abundance are shown here, with full results in Fig. S20. Sampling time points are on the *x*-axis and CLR-transformed abundance values are on the *y*-axis. Mean abundance values for day 0 and day 21 are provided for reference. *P* values are FDR-adjusted for multiple hypothesis test correction and results with *p* ≤ 0.05 considered significant.

At the phylum level, significant shifts in community proportions were observed in the subgingival and supragingival plaque. No statistically significant trends were observed at the phylum level in the tongue or salivary samples over the gingivitis induction period. From day 0 to day 21 after arresting OHP, the phylum-level dynamics were driven by increases in Bacteroidota (21.63% to 40.12% in subgingival and 18.78% to 32.78% in supragingival plaque) and Fusobacteriota (10.55% to 21.47% in subgingival and 9.96% to 18.86% in supragingival plaque) and decreases in Bacillota (34.63% to 24.48% in subgingival plaque), Pseudomonadota (21.29% to 8.42% in subgingival and 26.07% to 14.12% in supragingival plaque), and Actinomycetota (9.09% to 2.84% in subgingival and 10.30% to 5.24% in supragingival plaque). Several lower-abundance phyla (Spirochaetota, Campylobacterota, and Patescibacteria) also demonstrated significantly increasing trends, while two others (Desulfobacterota and Cyanobacteria) demonstrated significantly decreasing trends in the tooth plaque samples (Fig. 4, File S1). The most abundant genera with significantly increasing or decreasing community proportions are shown in Fig. 5. Before the suspension of OHP, the most abundant genera across all subjects in the subgingival plaque samples were *Streptococcus* (17.89%), *Neisseria* (9.58%), *Prevotella* (8.92%), *Fusobacterium* (8.57%), and *Veillonella* (6.33%).

After three weeks without OHP, the subgingival distribution shifted to *Prevotella* (21.00%), *Fusobacterium* (17.38%), *Capnocytophaga* (6.73%), *Veillonella* (4.85%), and *Porphyromonas* (4.54%). Supragingival results mirrored this closely, with the most abundant genera at day 0 being *Streptococcus* (17.93%), *Neisseria* (12.82%), *Fusobacterium* (7.47%), *Prevotella* (6.84%), and *Veillonella* (6.83%). After gingivitis induction, that ranking changed to *Fusobacterium* (12.87%), *Prevotella* (12.17%), *Veillonella* (10.58%), *Capnocytophaga* (10.48%), *Leptotrichia* (5.99%), and *Neisseria* (4.96%).

The salivary genus composition was considerably more stable, with the most abundant genera at day 0 being *Veillonella* (22.71%), *Prevotella* (21.04%), *Neisseria* (10.51%), *Streptococcus* (10.33%), and *Fusobacterium* (7.75%). By day 21, the rank-order of genera remained the same at *Veillonella* (22.48%), *Prevotella* (18.14%), *Neisseria* (10.09%), *Streptococcus* (10.26%), and *Fusobacterium* (7.98%).

The genus-level composition of the tongue was similarly stable, with most abundant genera at day 0: *Prevotella* (20.17%), *Veillonella* (17.99%), *Streptococcus* (10.95%), *Neisseria* (10.05%), and *Fusobacterium* (8.35%). By day 21 the ranking changed to *Prevotella* (23.15%), *Veillonella* (17.83%), *Fusobacterium* (11.05%), *Streptococcus* (7.64%), and *Neisseria* (6.59%). In the tongue samples, exactly one CLR-transformed result was statistically significant: an increase of the genus *Aggregatibacter* from 0.01% to 0.09% mean relative abundance over the induction phase, representing a 6.6-fold increase. The relative genus-level taxonomic stability of tongue and saliva samples is supported by the lower within-subject and between-subject beta diversity scores for samples from these sites. This finding underscores the importance of carefully assessing if saliva is an appropriately sensitive sample type to detect and track gingivitis and other oral health complications, and suggests subgingival and supragingival plaque samples could be more likely to capture bacterial community shifts.

Our results capture bacterial dynamics across every major human oral microbial clade, and a comprehensive walkthrough of the abundance dynamics is presented in the Supplementary Information. Key abundance shifts to highlight include the increase of *Neisseria* for the first two weeks after suspension of OHP followed by a rapid decline, the collapse of the genera *Haemophilus*, decreasing 30-fold from 3.41% to 0.11% in subgingival plaque; *Rothia*, decreasing 20-fold from 1.24% to 0.06% on average in subgingival plaque; and a similar 12-fold subgingival decrease in *Corynebacterium* durum from a mean of 0.45% to 0.04%, as well as a nearly 5-fold subgingival increase in *Alloprevotella* and 8-fold increase in *A. tannerae* specifically, and a statistically significant increase in *Cardiobacterium valvarum* in both tooth plaque environments (File S1).

### Dynamics within Socransky’s subgingival complexes after suspension of oral health practices

Given their historically cited role in periodontal health and disease, it is important to highlight the dynamics of the members of Socransky’s subgingival complexes after suspension of OHP [44]. Table 1 shows the distribution of Socransky’s complexes in the subgingival samples. No sequences classifying to members of the purple complex were observed. Among members of the green complex, *C. gingivalis* showed the only significant abundance trend, a decrease over the induction of gingivitis.

**Table 1:** Members of Socransky’s complexes after three weeks of oral hygiene practice suspension.

Complex	Taxonomic Group	Number of ASVs	Subgingival Mean Reads (%)	
			Day 0	Day 21
Red	*Treponema denticola*	4	0.205	0.191
Red	*Tannerella forsythia*	3	0.095	0.061
Red	*Porphyromonas gingivalis*	2	0.005	0.112
Orange	*Fusobacterium*^a^	471	8.565	17.383
Orange	*Prevotella intermedia*	12	1.453	1.775
Orange	*Prevotella nigrescens*^a^	44	0.875	2.988
Orange	*Campylobacter gracilis*	3	0.084	0.098
Orange	*Campylobacter showae*	6	0.058	0.144
Orange	*Eubacterium nodatum*	2	0.01	0.003
Orange	*Campylobacter rectus*	0	0	0
Orange	*Streptococcus constellatus*	0	0	0
Green	*Capnocytophaga gingivalis*^a^	13	0.849	0.335
Green	*Capnocytophaga sputigena*	6	0.681	1.356
Green	*Capnocytophaga ochracea*	8	0.023	0.046
Green	*Campylobacter concisus*	7	0.011	0.014
Green	*A. actinomycetemcomitans*	0	0	0
Purple	*Veillonella parvula*	0	0	0
Purple	*Schaalia odontolyticus*	0	0	0
Purple	*Selenomonas noxia*	0	0	0
Yellow	*Streptococcus*^a^	356	17.886	3.908
Blue	*Actinomyces*^a^	226	4.341	1.041

^a^Indicates that this taxonomic group’s abundance increase/decrease was statistically significant (non-zero slope) at *p* ≤ 0.05 after CLR transformation.

The members of the red complex demonstrated abundance shifts during early gingivitis onset. None of the shifts in red complex members were statistically significant across the 3-week gingivitis induction period, however known periopathogen *T. denticola* saw a small decrease in mean subgingival abundance between day 0 and 21, *T. forsythia* saw its abundance decrease by a third, and *P. gingivalis* saw a 22-fold increase while still remaining very low abundance as a proportion of the overall community.

The yellow and blue complexes both saw significant decreases in their relative community abundance during gingivitis induction. The yellow complex is populated by members of *Streptococcus*, which had three significantly decreasing ASVs found in three distinct clades for this gene region (Fig. S8). The blue complex, which is comprised of *Actinomyces*, also had three significantly decreasing ASVs: two belonged to a clade containing the species *A. naeslundii*, *A. oris*, and *A. viscosus* and the third significant ASV belonging to a clade with *A. gerencseriae* (Fig. S6).

The orange complex showed the most consistent dynamics, with all but one member (*Eubacterium nodatum*) seeing increases. However, only *Fusobacterium* and *Prevotella nigrescens* had statistically significant increases. Although no ASVs were classified as *C. rectus*, the gene region tree reveals little to no nucleotide differentiation between *C. rectus* and *C. showae* in the V4-V5 region (Fig. S13). No ASVs were classified down to *Streptococcus constellatus*, and none of the significantly increasing or decreasing ASVs placed near the *S. constellatus* reference sequence within the *Streptococcus* reference tree (Fig. S8), though this gene region contains insufficient nucleotide diversity to confidently distinguish most streptococcal species (see Supplementary Information section on “Phylogenetic and taxonomic resolution” for additional discussion).

## Discussion

Here we report a cross-sectional and longitudinal analysis of bacterial succession in the supragingival, subgingival, tongue and salivary microbiome, which occurs during the development of gingivitis and its resolution. This study was conducted as an extension of a single-arm clinical trial that characterized oral and circulatory polymorphonuclear neutrophils during the induction and resolution of experimental gingivitis [25]. The EGM constituted a three-week gingivitis induction phase during which 15 study participants discontinued all forms of oral hygiene (brushing, flossing, etc.) which was followed by a final recovery phase, that included OHP resumption for 2 more weeks. Overall, we identified trends in broad taxonomic groups (Figs. 4, 5) as well as trends in specific amplicon sequence variants (Figs. 3, S1–S19) during the early onset of, and recovery from, gingivitis.

**Fig. 6:**
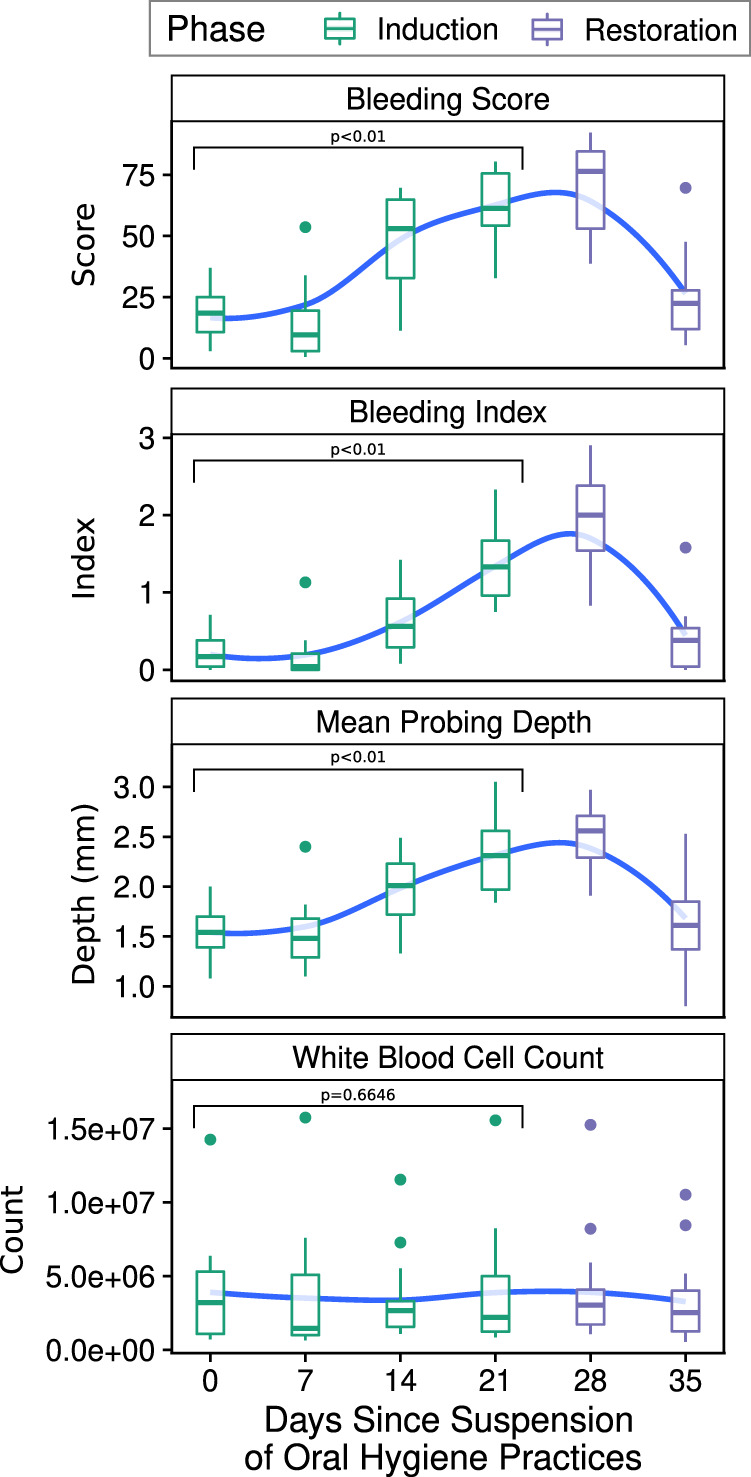
Indicators of periodontal health measured in subjects. Boxplots of indicators of periodontal health measured in ref. [25], across time. Boxplots show the median, 1st, and 3rd quartiles and 1.5 times the inter-quartile range, and LOESS smoothed regression lines are shown in blue to highlight trends. Indicated *p* value assesses the significance of the slope over the induction phase.

In particular, disease onset was characterized by a significant shift in individual taxonomic groups, but no significant whole community shifts detected by alpha or beta diversity measures. Both alpha and beta diversity results support that the salivary and tongue microbial composition are more stable over time and consistent between individuals than the subgingival and supragingival plaque in period after suspension of OHP. After OHP were resumed, relative abundance of the majority of taxonomic groups moved back to the baseline, in step with the immunological and periodontal measures published in Wellappulli et al. (Figs. 4–6, S20). For example in that publication, oral and blood polymorphonuclear neutrophils with cluster of differentiation (CD) markers CD11b and CD63 were found to decrease over gingivitis induction but returned to baseline after restoration [25]. Overall white blood cell count, it should be noted, did not significantly change over the gingivitis induction period (Fig. 6).

### Orange, yellow, blue complexes most dynamic subgingival complexes after 3 weeks without OHP

The microbial complexes described by Socransky et al. are reported to be important contributors to periodontal health and disease. In the red complex, *P. gingivalis* shows signs of an increase from a very low abundance state that were not statistically significant across all subjects, while the other two members of the complex, *T. denticola* and *T. forsythia*, saw decreases in their mean abundance (Table 1). Relative proportions of these organisms are generally higher in periodontal disease [28, 45], but the statistically non-significant results here suggest the role of the red complex may be limited in early gingivitis. In a recent EGM study that analyzed subgingival samples with marker gene sequencing, *Porphyromonas*, *Tannerella*, and *Treponema* all increased over gingivitis induction [10], consistent with genus-level results presented here (Fig. S20).

In the orange complex, an increase of the diverse genus *Fusobacterium* and the species *P. nigrescens* are the only two statistically significant results. Although not explicitly detected, it is possible that *C. rectus* is present and it is being misclassified as *C. showae* under our experimental parameters. On the other hand, no significant species or ASVs were detected near *S. constellatus* on the reference *Streptococcus* tree (Fig. S8). These results align with results in Bamashmous et al. [10], with the exception of *Fusobacterium* which showed a more consistent increase in gingivitis induction with our cohort and experimental parameters.

From the streptococcal tree, the yellow complex had four groups that decreased over induction of gingivitis: *S. mitis/oralis/infantis*, *S. sanguinis*, *S. parasanguinis/australis*, and *S. salivarius/vestibularis/thermophilus* (Fig. S8). In their work, Bamashmous et al. [10] also reported a significant drop in the streptococcal population. In that study, the authors suggest that *Streptococcus* are characteristic organisms of their “slow responder” group that experienced inflammatory and bacterial changes on a marked delay from other “high” and “low” responders. In Wellappuli et al. [25], the article first analyzing the inflammatory profile of the samples presented in this work, the samples were classified into high and low inflammatory levels, but no slow responders were specifically identified.

In Socransky’s blue complex, *Actinomyces* had three ASVs in two groups of species that decreased on aggregate: two ASVs in a group containing *A. oris/naeslundii/viscosus/johnsonii* and one ASV in a group with *A. gerencseriae/israelii* (Fig. S6). Our results in this genus most closely match the low responders group of [10].

### Restarting oral hygiene practices returns community to baseline

Our results show that the reestablishment of OHP after an extended period of suspension allows the bacterial community to revert to a state similar to that observed in the baseline. Alpha diversity values increase over time during induction of gingivitis, but decrease to baseline level after a “dental cleaning” procedure and resumption of daily OHP. The majority of taxonomic groups that were disrupted return to near the baseline levels observed after the restoration phase (time point −7, pre-induction phase). The communities return to baseline (as shown by a return to pre-induction phase abundance ranges) largely by time point 28 (e.g., Fig. 4), and as the healthy community is re-established, subjects’ bleeding index, bleeding score, and mean pocket depth gradually return to their baseline one week later (time point 35) (Fig. 6) [25]. These results are in line with the return to baseline characteristics and community composition observed in Bamashmous et al. [10].

### Limitations and considerations

This study provides a detailed and thorough descriptive analysis of the bacterial taxonomic shifts during gingivitis induction and recovery in four oral sites. The amplicon sequencing method allows for the characterization of microbial communities but comes with several important limitations. The first is that the amplicon abundance counts are compositional data, so the true abundance is virtually unknowable without deeper experimental validation such as quantitative PCR and we rely instead on relative proportions to draw inferences (in the case of the CLR transform, abundances are proportions relative to the geometric mean abundance of all taxa). A more thorough discussion on the implications and interpretation of compositional data is in the Supplementary Information. Another limitation of amplicon sequencing is that the taxonomic resolution provided by a gene fragment differs from group to group. Taxonomic classification methods like naïve Bayes are often unable to differentiate which species a sequence originated from, and this and other considerations on taxonomic resolution are discussed further in the Supplementary Information. Finally, our cohort of fifteen individuals was sufficient to highlight numerous taxonomic shifts consistent across those subjects, but given the high-dimensional and sparse nature of amplicon sequence-derived microbial count data, future experimental gingivitis investigations should consider a larger sample size to increase statistical power.

## Conclusions

We have identified broad microbial taxonomic groups and amplicon sequence variants that shift in proportion in the oral community in response to a suspension of OHP. The temporal correlations presented in this work may help identify bacterial taxa that are clinically relevant to gingivitis and periodontal disease. Our microbial sequence variants, mapped to the eHOMD reference set, contribute new candidates for these purposes and can help guide future studies which seek to establish stronger causal links. These results underscore the diversity of bacterial taxa that respond to oral hygiene practices, with significant dynamics spread across ten phyla (Fig. 3, File S1). Modern high-resolution taxonomic studies show that there are many organisms beyond Socransky’s complexes, particularly in the rare biosphere, that have strong associations with inflammation and disease but remain to be thoroughly characterized (for example, certain members of Absconditabacteriales (SR1) and Patescibacteria). Our results were largely consistent with previous EGM studies and help further elucidate the complex bacterial dynamics in gingivitis and its resolution.

## Supplementary information

Figure S1

Figure S2

Figure S3

Figure S4

Figure S5

Figure S6

Figure S7

Figure S8

Figure S9

Figure S10

Figure S11

Figure S12

Figure S13

Figure S14

Figure S15

Figure S16

Figure S17

Figure S18

Figure S19

Figure S20

Figure S21

Supplemental File 1

Supplemental Information

## Data Availability

Scripts, data, and computational notebooks for this experiment are available on GitHub at https://github.com/mwhall/Experimental_Gingivitis/. Raw sequences are deposited in the European Bioinformatics Institute’s Sequence Read Archive (EBI SRA) at project accession PRJEB37207.

## References

[1] AlJehani YA. Risk factors of periodontal disease: review of the literature. Int J Dent. 2014: p. 1–9. 10.1155/2014/182513. 10.1155/2014/182513 PMC4055151 24963294

[2] Nibali L, Farias B, Vajgel A, Tu Y, Donos N (2013). Tooth loss in aggressive periodontitis. J Dent Res.

[3] Tumolo AT (2013). Effects of periodontitis. J Am Dent Assoc.

[4] Könönen E, Gursoy M, Gursoy U (2019). Periodontitis: a multifaceted disease of tooth-supporting tissues. J Clin Med.

[5] Löe H, Theilade E, Jensen SB (1965). Experimental gingivitis in man. J Periodontol.

[6] Grant MM, Creese AJ, Barr G, Ling MR, Scott AE, Matthews JB (2010). Proteomic analysis of a noninvasive human model of acute inflammation and its resolution: The twenty-one day gingivitis model. J Proteome Res.

[7] Huang S, Li R, Zeng X, He T, Zhao H, Chang A (2014). Predictive modeling of gingivitis severity and susceptibility via oral microbiota. ISME J.

[8] Kistler JO, Booth V, Bradshaw DJ, Wade WG (2013). Bacterial community development in experimental gingivitis. PLoS ONE.

[9] Lee A, Ghaname CB, Braun TM, Sugai JV, Teles RP, Loesche WJ (2012). Bacterial and salivary biomarkers predict the gingival inflammatory profile. J Periodontol.

[10] Bamashmous S, Kotsakis GA, Kerns KA, Leroux BG, Zenobia C, Chen D (2021). Human variation in gingival inflammation. Proc Natl Acad Sci.

[11] Offenbacher S, Barros SP, Paquette DW, Winston JL, Biesbrock AR, Thomason RG (2009). Gingival transcriptome patterns during induction and resolution of experimental gingivitis in humans. J Periodontol.

[12] Abusleme L, Dupuy AK, Dutzan N, Silva N, Burleson JA, Strausbaugh LD (2013). The subgingival microbiome in health and periodontitis and its relationship with community biomass and inflammation. ISME J.

[13] Galimanas V, Hall MW, Singh N, Lynch MDJ, Goldberg M, Tenenbaum H, et al. Bacterial community composition of chronic periodontitis and novel oral sampling sites for detecting disease indicators. Microbiome. 2, 2014. 10.1186/2049-2618-2-32. 10.1186/2049-2618-2-32 PMC4164120 25225610

[14] Lenartova M, Tesinska B, Janatova T, Hrebicek O, Mysak J, Janata J, et al. The oral microbiome in periodontal health. Front Cell Infect Microbiol. 2021;11:219. 10.3389/fcimb.2021.629723 PMC8019927 33828997

[15] Marotz C, Molinsky R, Martino C, Bohn B, Roy S, Rosenbaum M (2022). Early microbial markers of periodontal and cardiometabolic diseases in origins. NPJ Biofilms Microbiomes.

[16] Szafranski SP, Wos-Oxley ML, Vilchez-Vargas R, Jáuregui R, Plumeier I, Klawonn F (2014). High-resolution taxonomic profiling of the subgingival microbiome for biomarker discovery and periodontitis diagnosis. Appl Environ Microbiol.

[17] Tu Q, Yu H, He Z, Deng Y, Wu L, Nostrand JDV, et al. GeoChip 4: a functional gene-array-based high-throughput environmental technology for microbial community analysis. Mol Ecol Resources. 2014. 10.1111/1755-0998.12239. 10.1111/1755-0998.12239 24520909

[18] Curtis MA, Diaz PI, Van Dyke TE (2020). The role of the microbiota in periodontal disease. Periodontology 2000.

[19] Huang S, He T, Yue F, Xu X, Wang L, Zhu P (2021). Longitudinal multi-omics and microbiome meta-analysis identify an asymptomatic gingival state that links gingivitis, periodontitis, and aging. mBio.

[20] Nowicki EM, Shroff R, Singleton JA, Renaud DE, Wallace D, Drury J, et al. Microbiota and metatranscriptome changes accompanying the onset of gingivitis. mBio. 2018, 9. 10.1128/mbio.00575-18. 10.1128/mBio.00575-18 PMC5904416 29666288

[21] Dewhirst FE, Chen T, Izard J, Paster BJ, Tanner ACR, Yu W-H (2010). The human oral microbiome. J Bacteriol.

[22] Human Microbiome Project Consortium (2012). Structure, function and diversity of the healthy human microbiome. Nature.

[23] Wade WG (2013). The oral microbiome in health and disease. Pharmacol Res.

[24] Ximénez-Fyvie LA, Haffajee AD, Socransky SS (2000). Microbial composition of supra- and subgingival plaque in subjects with adult periodontitis. J Clin Periodontol.

[25] Wellappuli N, Fine N, Lawrence H, Goldberg M, Tenenbaum H, Glogauer M (2017). Oral and blood neutrophil activation states during experimental gingivitis. JDR Clin Transl Res.

[26] Löe H, Silness J (1963). Periodontal disease in pregnancy I. prevalence and severity. Acta Odontol Scand.

[27] Löe H (1967). The gingival index, the plaque index and the retention index systems. J Periodontol.

[28] Griffen AL, Beall CJ, Campbell JH, Firestone ND, Kumar PS, Yang ZK (2011). Distinct and complex bacterial profiles in human periodontitis and health revealed by 16S pyrosequencing. ISME J.

[29] Könönen E, Wade WG (2015). Actinomyces and related organisms in human infections. Clin Microbiol Rev.

[30] Kreth J, Zhang Y, Herzberg MC (2008). Streptococcal antagonism in oral biofilms: Streptococcus sanguinis and Streptococcus gordonii interference with Streptococcus mutans. J Bacteriol.

[31] Zilm PS, Bagley CJ, Rogers AH, Milne IR, Gully NJ (2007). The proteomic profile of *Fusobacterium nucleatum* is regulated by growth pH. Microbiology.

[32] Coit P, Mumcu G, Ture-Ozdemir F, Unal AU, Alpar U, Bostanci N (2016). Sequencing of 16S rRNA reveals a distinct salivary microbiome signature in Behçet’s disease. Clin Immunol.

[33] Comeau AM, Douglas GM, Langille MGI. Microbiome helper: a custom and streamlined workflow for microbiome research. mSystems, 2, 2017. 10.1128/msystems.00127-16. 10.1128/mSystems.00127-16 PMC5209531 28066818

[34] Walters W, Hyde ER, Berg-Lyons D, Ackermann G, Humphrey G, Parada A, et al. Improved bacterial 16S rRNA gene (V4 and V4-5) and fungal internal transcribed spacer marker gene primers for microbial community surveys. mSystems, 2016: 1. 10.1128/msystems.00009-15. 10.1128/mSystems.00009-15 PMC5069754 27822518

[35] Bolyen E, Rideout JR, Dillon MR, Bokulich NA, Abnet CC, Al-Ghalith GA (2019). Reproducible, interactive, scalable and extensible microbiome data science using QIIME2. Nat Biotechnol.

[36] Callahan BJ, McMurdie PJ, Rosen MJ, Han AW, Johnson AJA, Holmes SP (2016). DADA2: High-resolution sample inference from Illumina amplicon data. Nat Methods.

[37] Pruesse E, Quast C, Knittel K, Fuchs BM, Ludwig W, Peplies J (2007). SILVA: a comprehensive online resource for quality checked and aligned ribosomal RNA sequence data compatible with ARB. Nucleic Acids Res.

[38] Van den Boogaart KG, Tolosana-Delgado R (2008). “compositions”: a unified R package to analyze compositional data. Comput Geosci.

[39] Bates D, Mächler M, Bolker B. Walker S. Fitting linear mixed-effects models using lme4. arXiv Preprint. arXiv. 2014;1406:5823.

[40] Kuznetsova A, Brockhoff PB, Christensen RH (2017). lmerTest package: tests in linear mixed effects models. J Stat Softw.

[41] Katoh K, Standley DM (2013). MAFFT multiple sequence alignment software version 7: improvements in performance and usability. Mol Biol Evol.

[42] Price MN, Dehal PS, Arkin AP (2010). FastTree 2–approximately maximum-likelihood trees for large alignments. PLoS One.

[43] Yu G (2020). Using ggtree to visualize data on tree-like structures. Curr Protoc Bioinform.

[44] Socransky S, Haffajee A, Cugini M, Smith C, Kent RL (1998). Microbial complexes in subgingival plaque. J Clin Periodontol.

[45] Rafiei M, Kiani F, Sayehmiri F, Sayehmiri K, Sheikhi A, Azodi MZ (2017). Study of Porphyromonas gingivalis in periodontal diseases: a systematic review and meta-analysis. Med J Islamic Repub Iran.

