# Correction: Suspension of oral hygiene practices highlights key bacterial shifts in saliva, tongue, and tooth plaque during gingival inflammation and resolution

**DOI:** 10.1038/s43705-023-00258-0

**Published:** 2023-05-31

**Authors:** Michael William Hall, Nimali Chandhema Wellappuli, Ruo Chen Huang, Kay Wu, David King Lam, Michael Glogauer, Robert Gerald Beiko, Dilani Braziunas Senadheera

**Affiliations:** 1 Faculty of Graduate Studies, Dalhousie University, Halifax, NS Canada; 2 Faculty of Dentistry, University of Toronto, Toronto, ON Canada; 3 McMaster University, Hamilton, ON Canada; 4 University of the Pacific, San Francisco, CA USA; 5 Faculty of Computer Science, Dalhousie University, Halifax, NS Canada; 6 Stony Brook University, School of Dental Medicine, Stony Brook, NY USA

**Keywords:** Microbiome, Biomarkers, Population dynamics

Correction to: *ISME Communications* 10.1038/s43705-023-00229-5, published online 25 March 2023

In this article the author name David King Lam was incorrectly written as David Kitping Lam.

The original article has been corrected.

