# Supplementary figures and images for: Suspension of oral hygiene practices highlights key bacterial shifts in saliva, tongue, and tooth plaque during gingival inflammation and resolution
Source: ISME Commun. 2023 Mar 25;3:23. doi: 10.1038/s43705-023-00229-5 (PMC10039884; doi:10.1038/s43705-023-00229-5)

# Spirochaetota

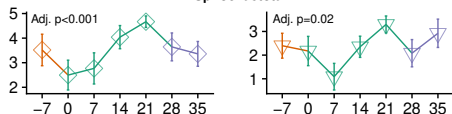

# Treponema

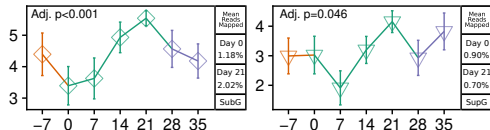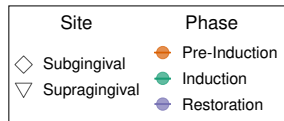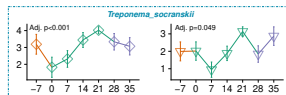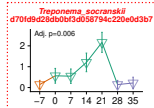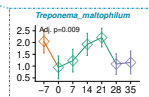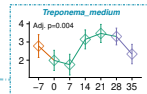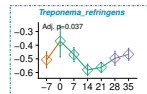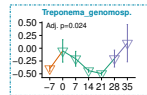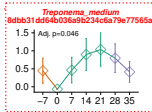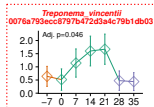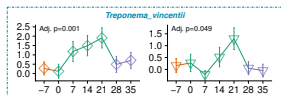

# Escherichia

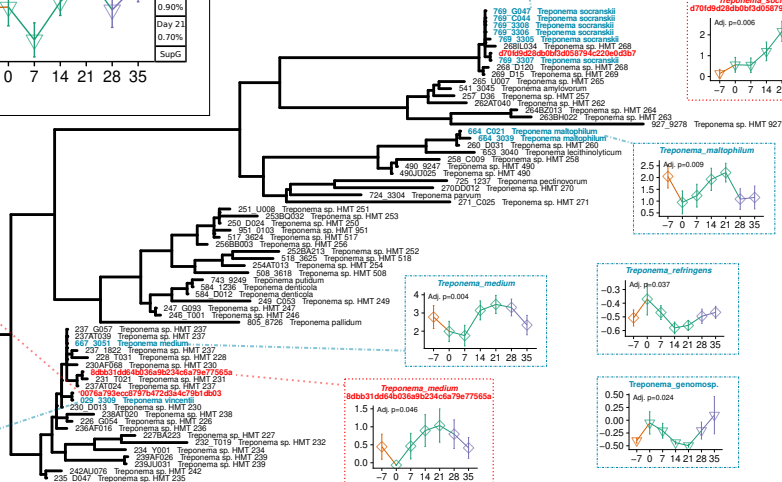

Supplement: Supplementary file 1 — Figure S1 [file 43705_2023_229_MOESM1_ESM.pdf]

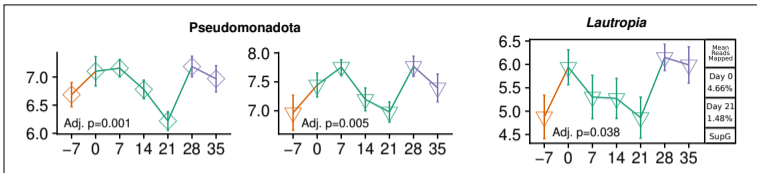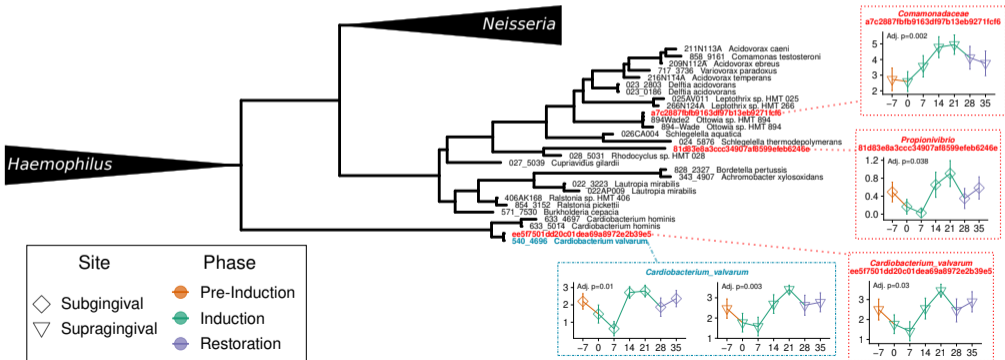

Supplement: Supplementary file 3 — Figure S3 [file 43705_2023_229_MOESM3_ESM.pdf]

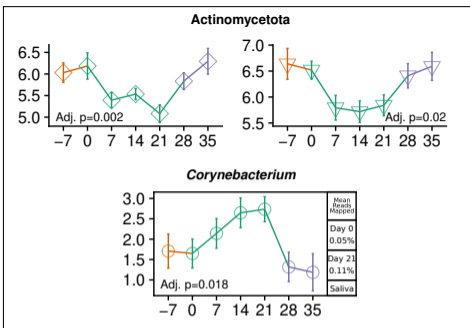

**Fretibacterium**

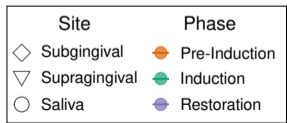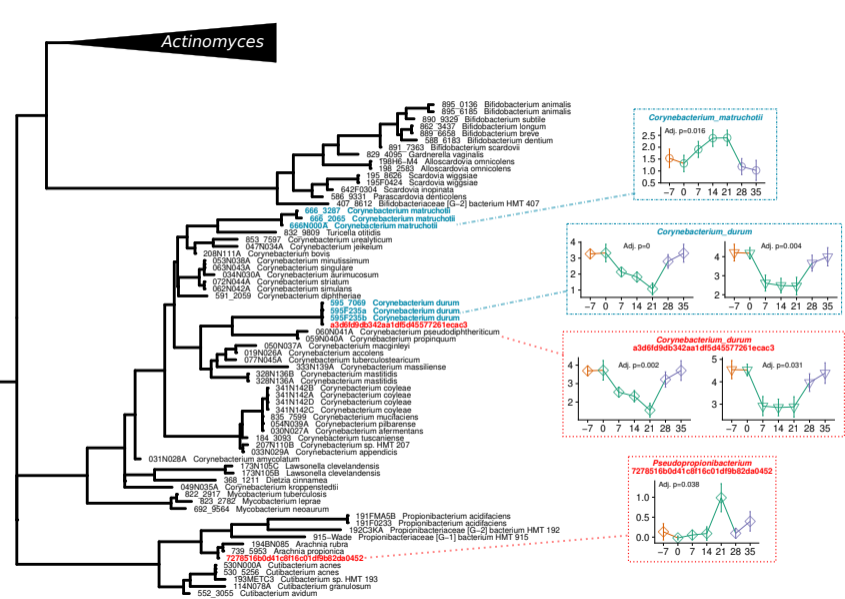

Supplement: Supplementary file 5 — Figure S5 [file 43705_2023_229_MOESM5_ESM.pdf]

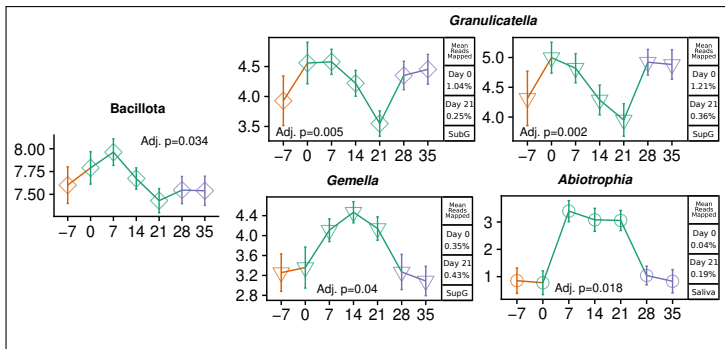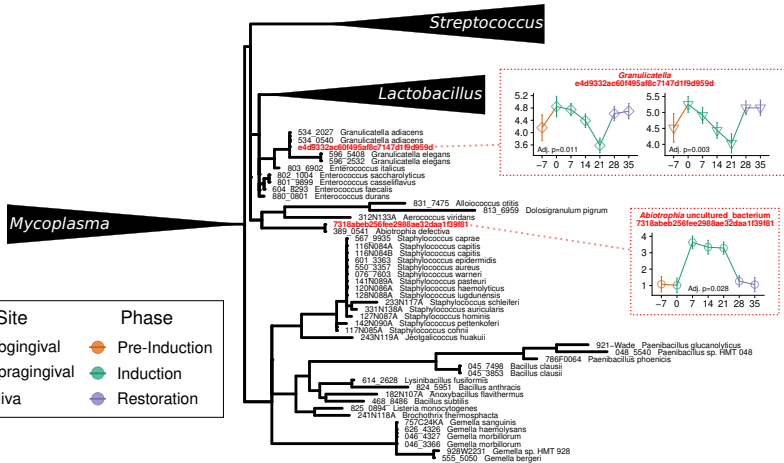

Supplement: Supplementary file 7 — Figure S7 [file 43705_2023_229_MOESM7_ESM.pdf]

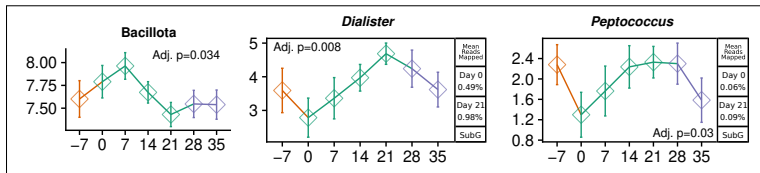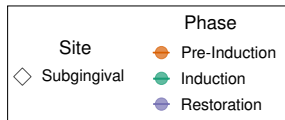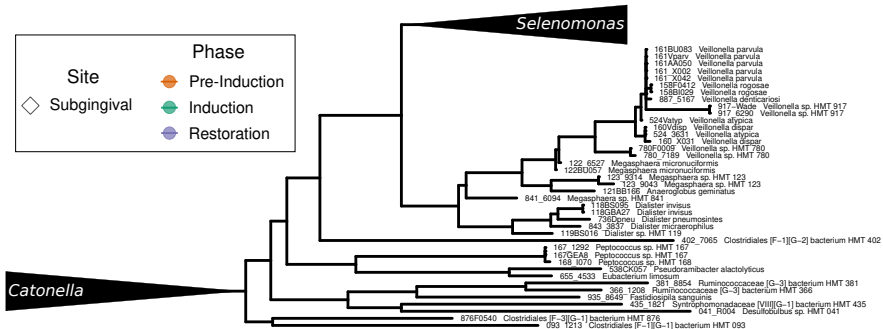

Supplement: Supplementary file 9 — Figure S9 [file 43705_2023_229_MOESM9_ESM.pdf]

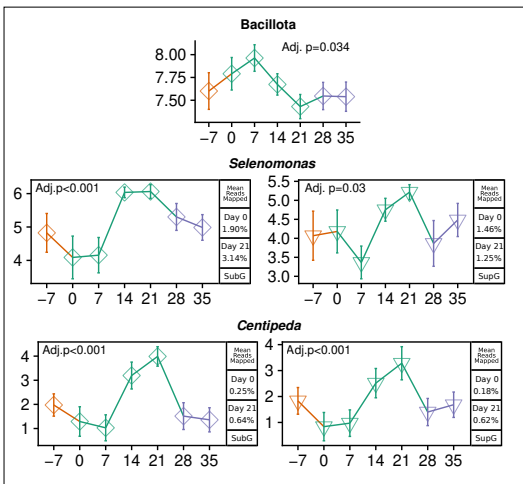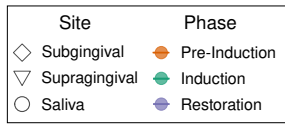

**Veillonella**

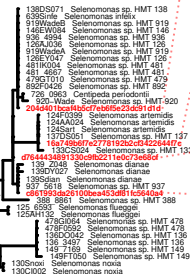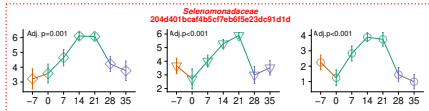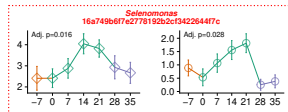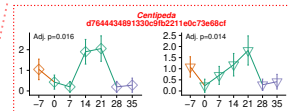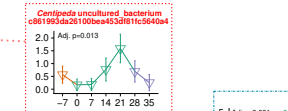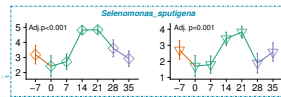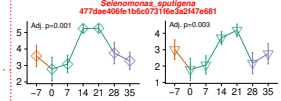

Supplement: Supplementary file 10 — Figure S10 [file 43705_2023_229_MOESM10_ESM.pdf]

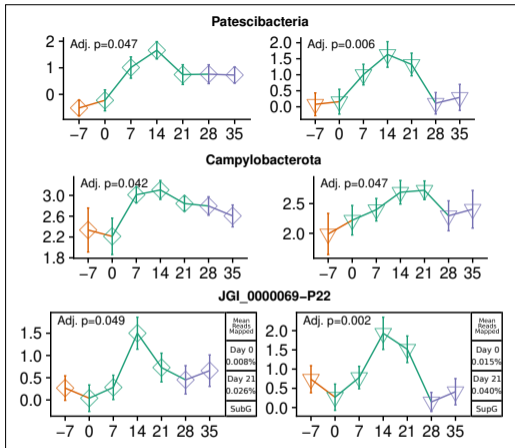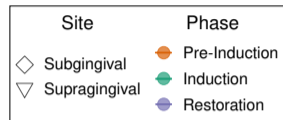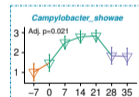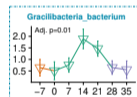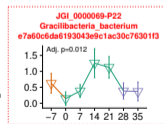

**Saccharibacteria**

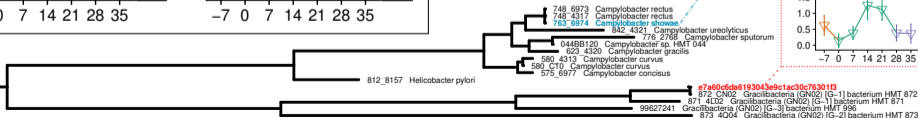

Supplement: Supplementary file 13 — Figure S13 [file 43705_2023_229_MOESM13_ESM.pdf]

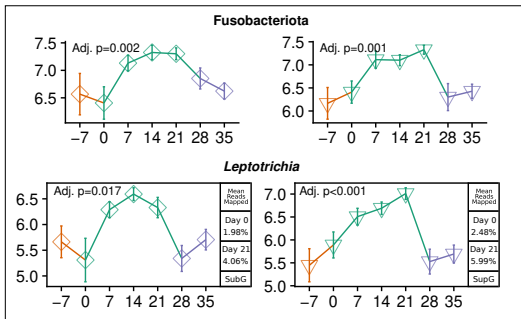

**Fusobacterium**

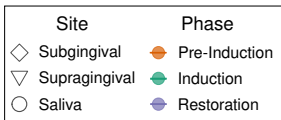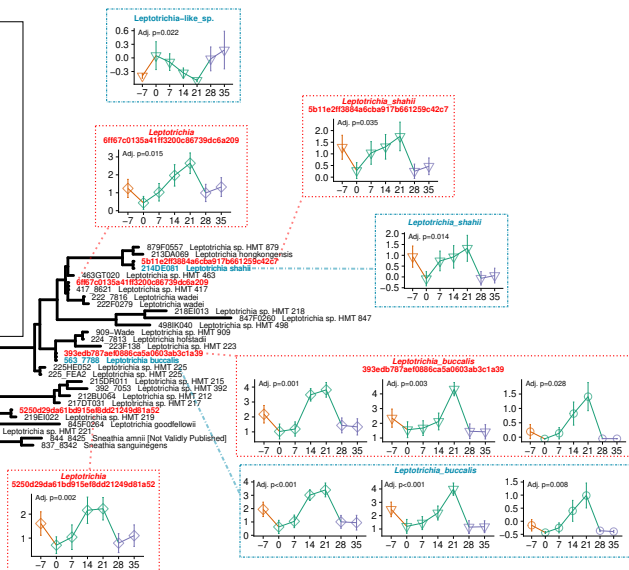

Supplement: Supplementary file 16 — Figure S16 [file 43705_2023_229_MOESM16_ESM.pdf]

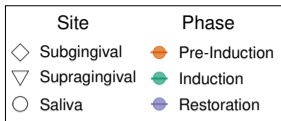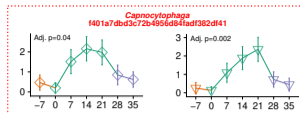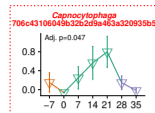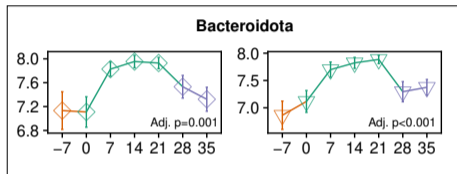

**Prevotellaceae**

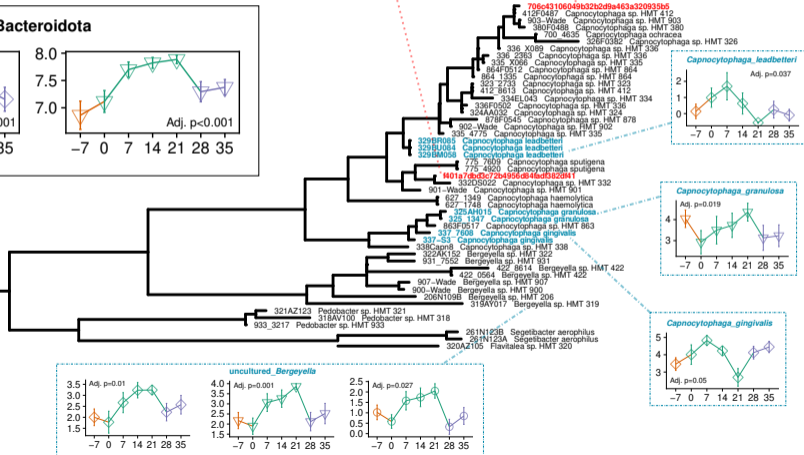

Supplement: Supplementary file 17 — Figure S17 [file 43705_2023_229_MOESM17_ESM.pdf]

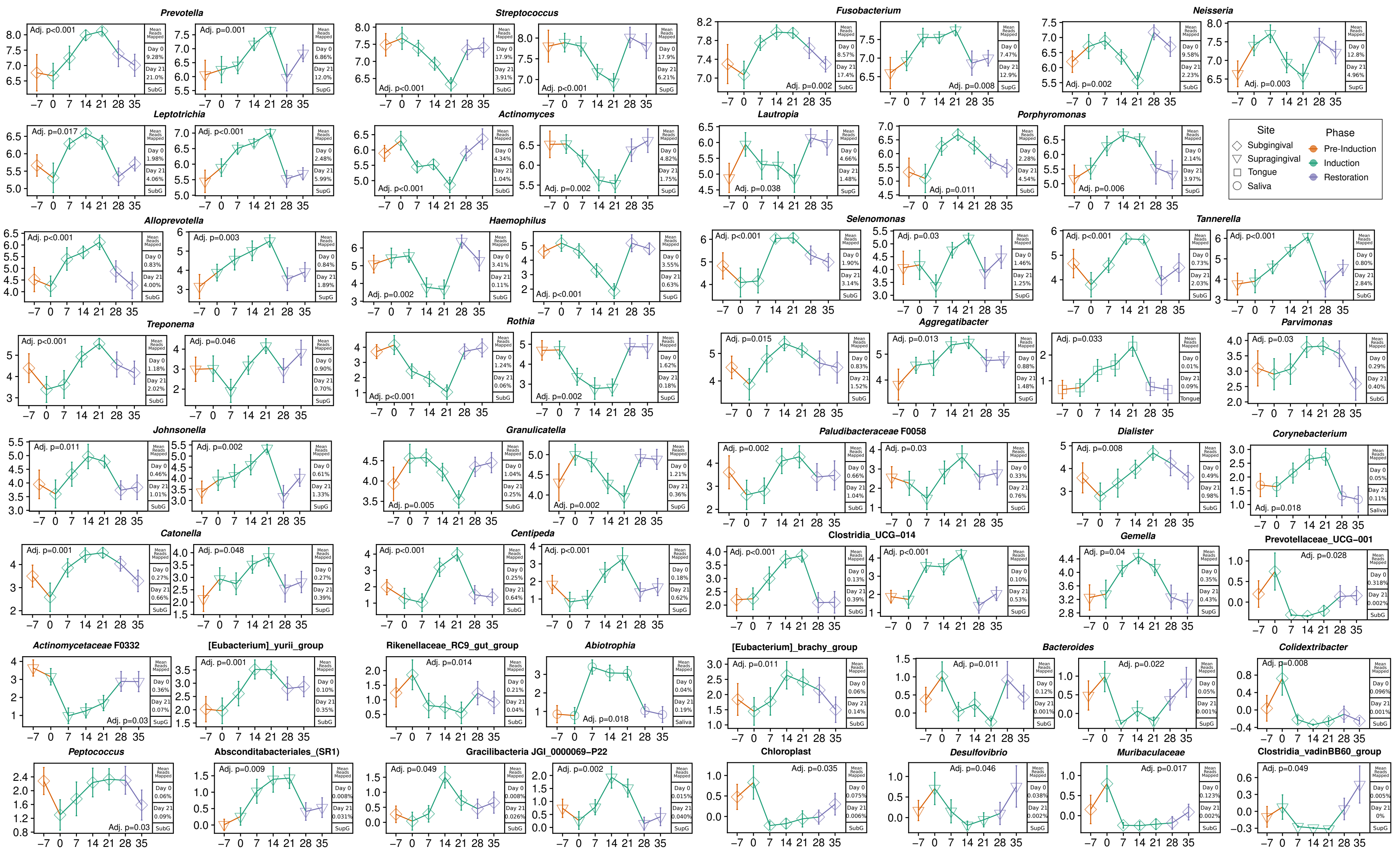

Supplement: Supplementary file 20 — Figure S20 [file 43705_2023_229_MOESM20_ESM.pdf]

Phase 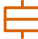 Pre-induction 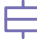 Induction 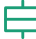 Restoration

Bray Curtis Dissimilarity

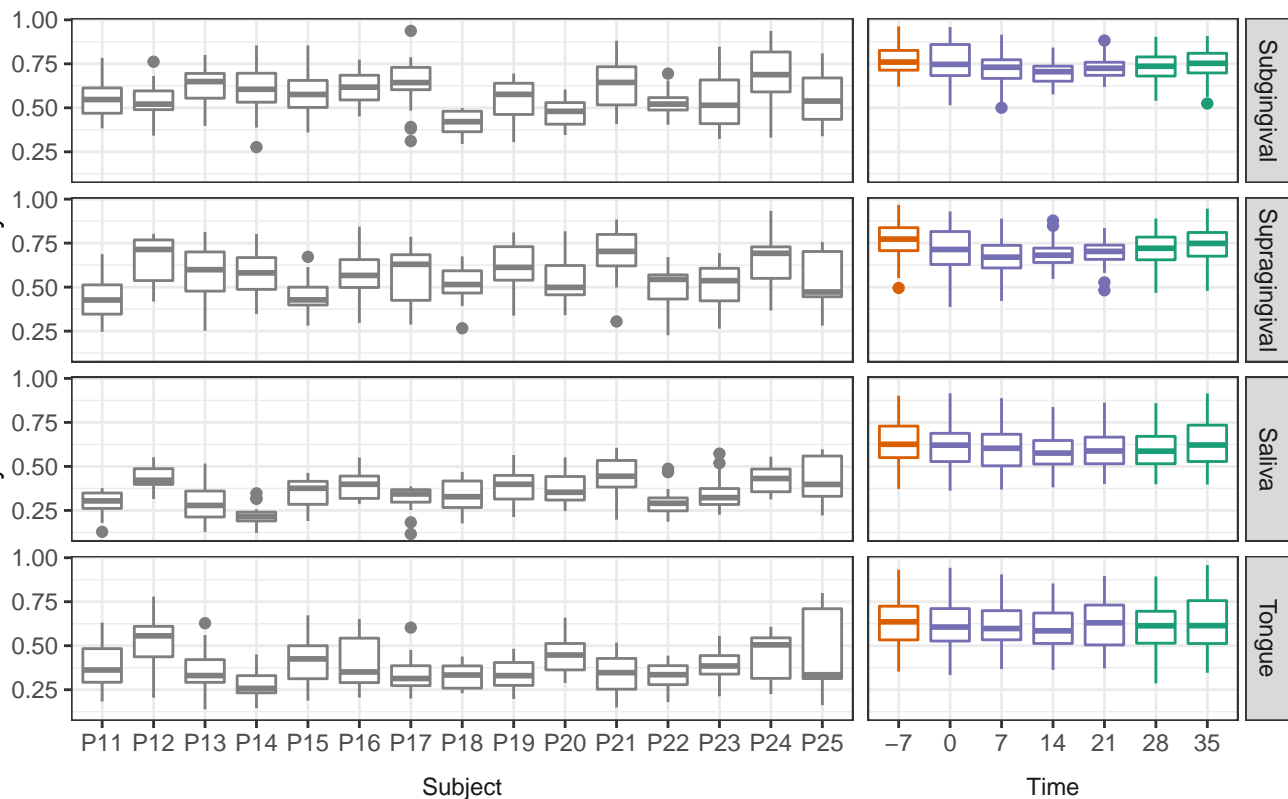

Supplement: Supplementary file 21 — Figure S21 [file 43705_2023_229_MOESM21_ESM.pdf]
