# Supplementary material for: Suspension of oral hygiene practices highlights key bacterial shifts in saliva, tongue, and tooth plaque during gingival inflammation and resolution
Source: ISME Commun. 2023 Mar 25;3:23. doi: 10.1038/s43705-023-00229-5 (PMC10039884; doi:10.1038/s43705-023-00229-5)
Supplement: Supplementary file 2 — Figure S2 [file 43705_2023_229_MOESM2_ESM.pdf]

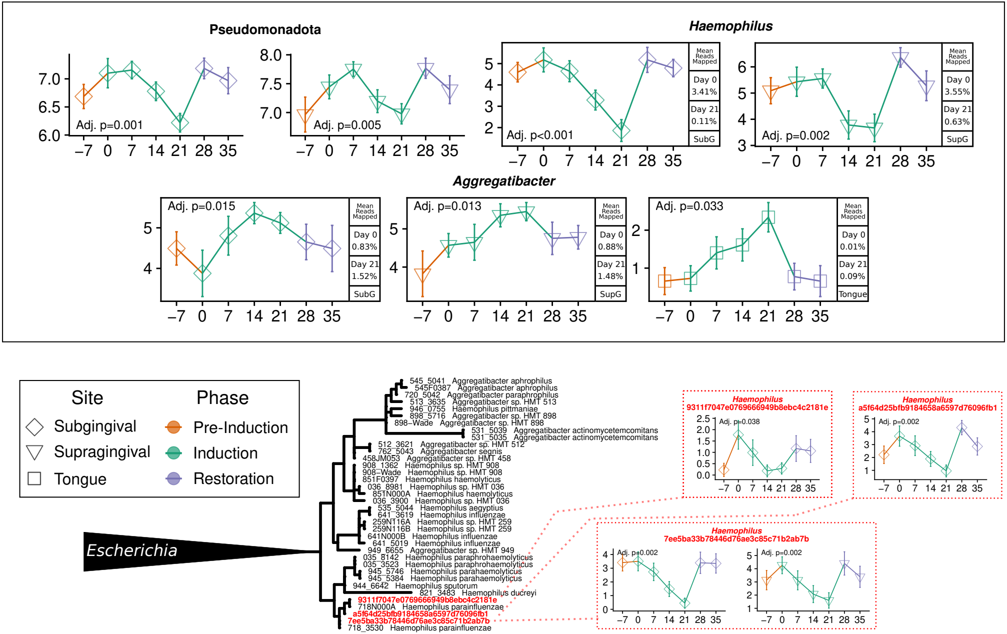
Pseudomonadota
Adj. p=0.001
Adj. p=0.005
Haemophilus
Mean Reads Mapped
Day 0 3.41%
Day 21 0.11%
SubG
Adj. p<0.001
Day 0 3.55%
Day 21 0.63%
SupG
Adj. p=0.002
Aggregatibacter
Adj. p=0.015
Day 0 0.83%
Day 21 1.52%
SubG
Adj. p=0.013
Day 0 0.88%
Day 21 1.48%
SupG
Adj. p=0.033
Day 0 0.01%
Day 21 0.09%
Tongue
Site
Subgingival
Supragingival
Tongue
Phase
Pre-Induction
Induction
Restoration
Escherichia
545_5041 Aggregatibacter aphrophilus
545F0387 Aggregatibacter aphrophilus
720_5042 Aggregatibacter paraphrophilus
513_3635 Aggregatibacter sp. HMT 513
946_0755 Haemophilus pittmaniae
898_5716 Aggregatibacter sp. HMT 898
898–Wade Aggregatibacter sp. HMT 898
531_5039 Aggregatibacter actinomycetemcomitans
531_5035 Aggregatibacter actinomycetemcomitans
512_3621 Aggregatibacter sp. HMT 512
762_5043 Aggregatibacter segnis
458JM053 Aggregatibacter sp. HMT 458
908_1362 Haemophilus sp. HMT 908
908–Wade Haemophilus sp. HMT 908
851F0397 Haemophilus haemolyticus
036_8981 Haemophilus sp. HMT 036
851N000A Haemophilus haemolyticus
036_3900 Haemophilus sp. HMT 036
535_5044 Haemophilus aegyptius
641_3619 Haemophilus influenzae
259N116A Haemophilus sp. HMT 259
259N116B Haemophilus sp. HMT 259
641N000B Haemophilus influenzae
641_5019 Haemophilus influenzae
949_6655 Aggregatibacter sp. HMT 949
035_8142 Haemophilus paraphrohaemolyticus
035_3523 Haemophilus paraphrohaemolyticus
945_5746 Haemophilus parahaemolyticus
945_5384 Haemophilus parahaemolyticus
944_6642 Haemophilus sputorum
821_3483 Haemophilus ducreyi
9311f7047e0769666949b8ebc4c2181e
718N000A Haemophilus parainfluenzae
a5f64d25bfb9184658a6597d76096fb1
7ee5ba33b78446d76ae3c85c71b2ab7b
718_3530 Haemophilus parainfluenzae
Haemophilus
9311f7047e0769666949b8ebc4c2181e
Adj. p=0.038
Haemophilus
a5f64d25bfb9184658a6597d76096fb1
Adj. p=0.002
Haemophilus
7ee5ba33b78446d76ae3c85c71b2ab7b
Adj. p=0.002
Adj. p=0.002
