# Supplementary material for: Suspension of oral hygiene practices highlights key bacterial shifts in saliva, tongue, and tooth plaque during gingival inflammation and resolution
Source: ISME Commun. 2023 Mar 25;3:23. doi: 10.1038/s43705-023-00229-5 (PMC10039884; doi:10.1038/s43705-023-00229-5)
Supplement: Supplementary file 4 — Figure S4 [file 43705_2023_229_MOESM4_ESM.pdf]

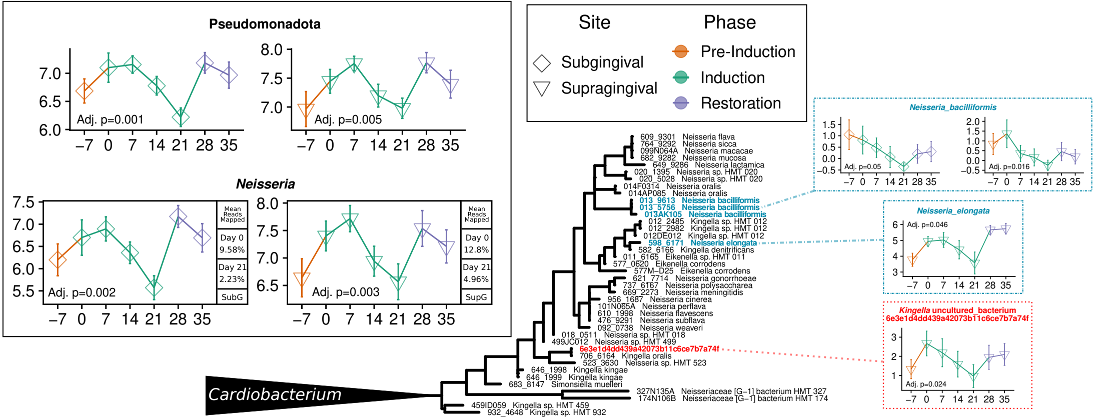

Pseudomonadota
Adj. p=0.001
Adj. p=0.005
Neisseria
Adj. p=0.002
Adj. p=0.003
Mean Reads Mapped
Day 0
9.58%
Day 21
2.23%
SubG
Mean Reads Mapped
Day 0
12.8%
Day 21
4.96%
SupG
−7 0 7 14 21 28 35
Site
Subgingival
Supragingival
Phase
Pre-Induction
Induction
Restoration
Neisseria_bacilliformis
Adj. p=0.05
Adj. p=0.016
Neisseria_elongata
Adj. p=0.046
Kingella uncultured_bacterium
6e3e1d4dd439a42073b11c6ce7b7a74f
Adj. p=0.024
609_9301 Neisseria flava
764_9292 Neisseria sicca
099N064A Neisseria macacae
682_9282 Neisseria mucosa
649_9286 Neisseria lactamica
020_1395 Neisseria sp. HMT 020
020_5028 Neisseria sp. HMT 020
014F0314 Neisseria oralis
014AP085 Neisseria oralis
013_9613 Neisseria bacilliformis
013_5756 Neisseria bacilliformis
013AK105 Neisseria bacilliformis
012_2485 Kingella sp. HMT 012
012_2982 Kingella sp. HMT 012
012DE012 Kingella sp. HMT 012
598_6171 Neisseria elongata
582_6166 Kingella denitrificans
011_6165 Eikenella sp. HMT 011
577_0620 Eikenella corrodens
577M–D25 Eikenella corrodens
621_7714 Neisseria gonorrhoeae
737_6167 Neisseria polysaccharea
669_2273 Neisseria meningitidis
956_1687 Neisseria cinerea
101N065A Neisseria perflava
610_1998 Neisseria flavescens
476_9291 Neisseria subflava
092_0738 Neisseria weaveri
018_0511 Neisseria sp. HMT 018
499JC012 Neisseria sp. HMT 499
6e3e1d4dd439a42073b11c6ce7b7a74f
706_6164 Kingella oralis
523_3630 Neisseria sp. HMT 523
646_1998 Kingella kingae
646_1999 Kingella kingae
683_8147 Simonsiella muelleri
327N135A Neisseriaceae [G–1] bacterium HMT 327
174N106B Neisseriaceae [G–1] bacterium HMT 174
459ID059 Kingella sp. HMT 459
932_4648 Kingella sp. HMT 932
Cardiobacterium
