# Supplementary material for: Suspension of oral hygiene practices highlights key bacterial shifts in saliva, tongue, and tooth plaque during gingival inflammation and resolution
Source: ISME Commun. 2023 Mar 25;3:23. doi: 10.1038/s43705-023-00229-5 (PMC10039884; doi:10.1038/s43705-023-00229-5)
Supplement: Supplementary file 6 — Figure S6 [file 43705_2023_229_MOESM6_ESM.pdf]

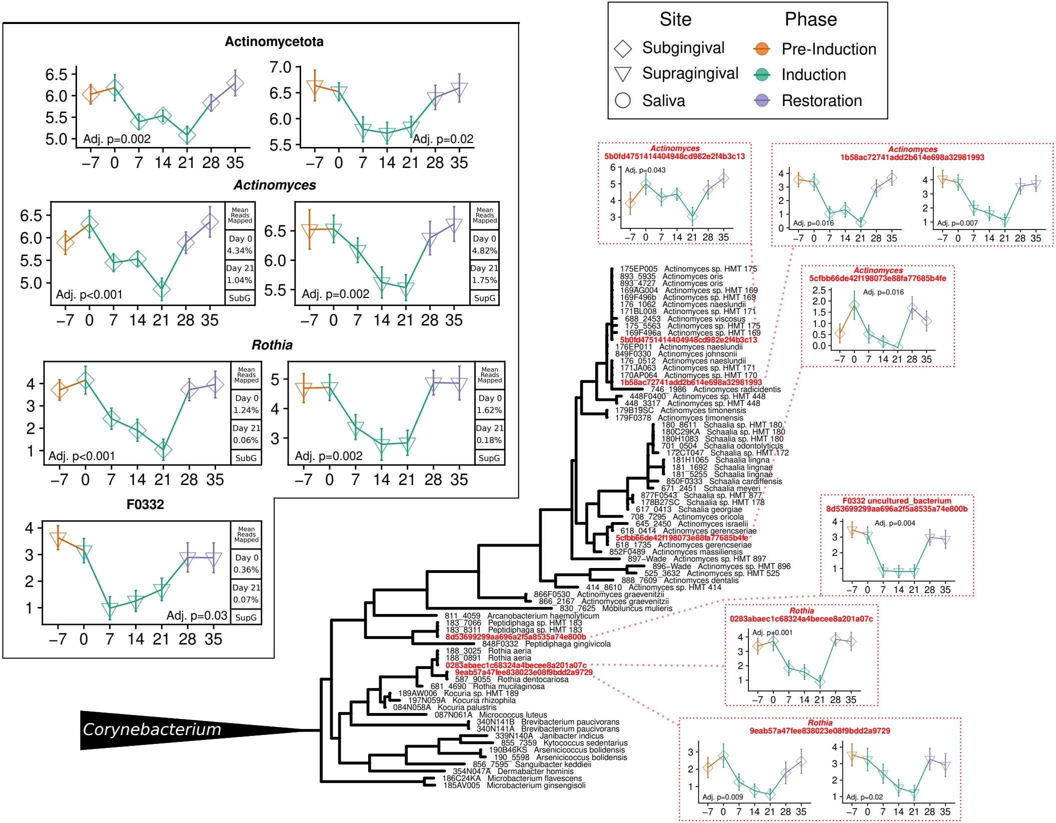

Site
Subgingival
Supragingival
Saliva
Phase
Pre-Induction
Induction
Restoration
Actinomycetota
Adj. p=0.002
Adj. p=0.02
Actinomyces
Adj. p<0.001
Adj. p=0.002
Mean Reads Mapped
Day 0 4.34%
Day 21 1.04%
SubG
Day 0 4.82%
Day 21 1.75%
SupG
Rothia
Adj. p<0.001
Adj. p=0.002
Day 0 1.24%
Day 21 0.06%
SubG
Day 0 1.62%
Day 21 0.18%
SupG
F0332
Adj. p=0.03
Day 0 0.36%
Day 21 0.07%
SupG
−7 0 7 14 21 28 35
Actinomyces
5b0fd4751414404948cd982e2f4b3c13
Adj. p=0.043
Actinomyces
1b58ac72741add2b614e698a32981993
Adj. p=0.016
Adj. p=0.007
Actinomyces
5cfbb66de42f198073e88fa77685b4fe
Adj. p=0.016
F0332 uncultured_bacterium
8d53699299aa696a2f5a8535a74e800b
Adj. p=0.004
Rothia
0283abaec1c68324a4becee8a201a07c
Adj. p=0.001
Rothia
9eab57a47fee838023e08f9bdd2a9729
Adj. p=0.009
Adj. p=0.02
175EP005 Actinomyces sp. HMT 175
893_5935 Actinomyces oris
893_4727 Actinomyces oris
169AG004 Actinomyces sp. HMT 169
169F496b Actinomyces sp. HMT 169
176_1062 Actinomyces naeslundii
171BL008 Actinomyces sp. HMT 171
688_2453 Actinomyces viscosus
175_5563 Actinomyces sp. HMT 175
169F496a Actinomyces sp. HMT 169
5b0fd4751414404948cd982e2f4b3c13
176EP011 Actinomyces naeslundii
849F0330 Actinomyces johnsonii
176_0512 Actinomyces naeslundii
171JA063 Actinomyces sp. HMT 171
170AP064 Actinomyces sp. HMT 170
1b58ac72741add2b614e698a32981993
746_1986 Actinomyces radicidentis
448F0400 Actinomyces sp. HMT 448
448_3317 Actinomyces sp. HMT 448
179B19SC Actinomyces timonensis
179F0378 Actinomyces timonensis
180_8611 Schaalia sp. HMT 180
180C29KA Schaalia sp. HMT 180
180H1083 Schaalia sp. HMT 180
701_0504 Schaalia odontolyticus
172CT047 Schaalia sp. HMT 172
181H1065 Schaalia lingna
181_1692 Schaalia lingnae
181_5255 Schaalia lingnae
850F0333 Schaalia cardiffensis
671_2451 Schaalia meyeri
877F0543 Schaalia sp. HMT 877
178B27SC Schaalia sp. HMT 178
617_0413 Schaalia georgiae
708_7295 Actinomyces oricola
645_2450 Actinomyces israelii
618_0414 Actinomyces gerencseriae
5cfbb66de42f198073e88fa77685b4fe
618_1735 Actinomyces gerencseriae
852F0489 Actinomyces massiliensis
897−Wade Actinomyces sp. HMT 897
896−Wade Actinomyces sp. HMT 896
525_3632 Actinomyces sp. HMT 525
888_7609 Actinomyces dentalis
414_8610 Actinomyces sp. HMT 414
866F0530 Actinomyces graevenitzii
866_2167 Actinomyces graevenitzii
830_7625 Mobiluncus mulieris
811_4059 Arcanobacterium haemolyticum
183_7066 Peptidiphaga sp. HMT 183
183_8311 Peptidiphaga sp. HMT 183
8d53699299aa696a2f5a8535a74e800b
848F0332 Peptidiphaga gingivicola
188_3025 Rothia aeria
188_0891 Rothia aeria
0283abaec1c68324a4becee8a201a07c
9eab57a47fee838023e08f9bdd2a9729
587_9055 Rothia dentocariosa
681_4690 Rothia mucilaginosa
189AW006 Kocuria sp. HMT 189
197N059A Kocuria rhizophila
084N058A Kocuria palustris
087N061A Micrococcus luteus
340N141B Brevibacterium paucivorans
340N141A Brevibacterium paucivorans
339N140A Janibacter indicus
855_7359 Kytococcus sedentarius
190B46KS Arsenicicoccus bolidensis
190_5598 Arsenicicoccus bolidensis
856_7595 Sanguibacter keddieii
354N047A Dermabacter hominis
186C24KA Microbacterium flavescens
185AV005 Microbacterium ginsengisoli
Corynebacterium
