# Supplementary material for: Suspension of oral hygiene practices highlights key bacterial shifts in saliva, tongue, and tooth plaque during gingival inflammation and resolution
Source: ISME Commun. 2023 Mar 25;3:23. doi: 10.1038/s43705-023-00229-5 (PMC10039884; doi:10.1038/s43705-023-00229-5)
Supplement: Supplementary file 8 — Figure S8 [file 43705_2023_229_MOESM8_ESM.pdf]

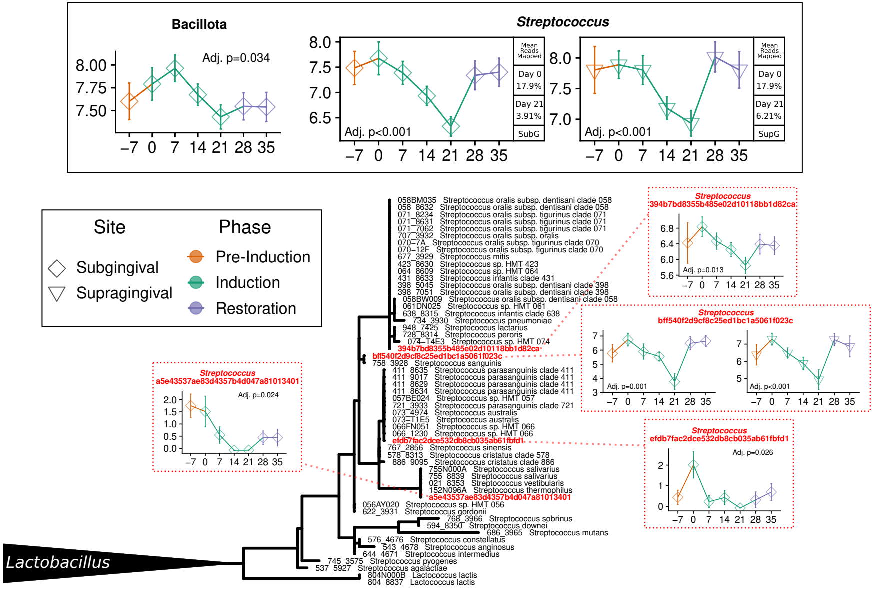

Bacillota
Adj. p=0.034
Streptococcus
Mean Reads Mapped
Day 0 17.9%
Day 21 3.91%
SubG
Adj. p<0.001
Day 0 17.9%
Day 21 6.21%
SupG
Site
Subgingival
Supragingival
Phase
Pre-Induction
Induction
Restoration
Streptococcus 394b7bd8355b485e02d10118bb1d82ca
Adj. p=0.013
Streptococcus bff540f2d9cf8c25ed1bc1a5061f023c
Adj. p=0.001
Adj. p<0.001
Streptococcus a5e43537ae83d4357b4d047a81013401
Adj. p=0.024
Streptococcus efdb7fac2dce532db8cb035ab61fbfd1
Adj. p=0.026
058BM035 Streptococcus oralis subsp. dentisani clade 058
058_8632 Streptococcus oralis subsp. dentisani clade 058
071_8234 Streptococcus oralis subsp. tigurinus clade 071
071_8631 Streptococcus oralis subsp. tigurinus clade 071
071_7062 Streptococcus oralis subsp. tigurinus clade 071
707_3932 Streptococcus oralis subsp. oralis
070–7A Streptococcus oralis subsp. tigurinus clade 070
070–12F Streptococcus oralis subsp. tigurinus clade 070
677_3929 Streptococcus mitis
423_8630 Streptococcus sp. HMT 423
064_8609 Streptococcus sp. HMT 064
431_8633 Streptococcus infantis clade 431
398_5045 Streptococcus oralis subsp. dentisani clade 398
398_7051 Streptococcus oralis subsp. dentisani clade 398
058BW009 Streptococcus oralis subsp. dentisani clade 058
061DN025 Streptococcus sp. HMT 061
638_8315 Streptococcus infantis clade 638
734_3930 Streptococcus pneumoniae
948_7425 Streptococcus lactarius
728_8314 Streptococcus peroris
074–T4E3 Streptococcus sp. HMT 074
394b7bd8355b485e02d10118bb1d82ca
bff540f2d9cf8c25ed1bc1a5061f023c
758_3928 Streptococcus sanguinis
411_8635 Streptococcus parasanguinis clade 411
411_9017 Streptococcus parasanguinis clade 411
411_8629 Streptococcus parasanguinis clade 411
411_8634 Streptococcus parasanguinis clade 411
057BE024 Streptococcus sp. HMT 057
721_3933 Streptococcus parasanguinis clade 721
073_4974 Streptococcus australis
073–T1E5 Streptococcus australis
066FN051 Streptococcus sp. HMT 066
066_1230 Streptococcus sp. HMT 066
efdb7fac2dce532db8cb035ab61fbfd1
767_2856 Streptococcus sinensis
578_8313 Streptococcus cristatus clade 578
886_9095 Streptococcus cristatus clade 886
755N000A Streptococcus salivarius
755_8839 Streptococcus salivarius
021_8353 Streptococcus vestibularis
152N096A Streptococcus thermophilus
a5e43537ae83d4357b4d047a81013401
056AY020 Streptococcus sp. HMT 056
622_3931 Streptococcus gordonii
768_3966 Streptococcus sobrinus
594_8350 Streptococcus downei
686_3965 Streptococcus mutans
576_4676 Streptococcus constellatus
543_4678 Streptococcus anginosus
644_4671 Streptococcus intermedius
745_3575 Streptococcus pyogenes
537_5927 Streptococcus agalactiae
804N000B Lactococcus lactis
804_8837 Lactococcus lactis
Lactobacillus
