# Supplementary material for: Suspension of oral hygiene practices highlights key bacterial shifts in saliva, tongue, and tooth plaque during gingival inflammation and resolution
Source: ISME Commun. 2023 Mar 25;3:23. doi: 10.1038/s43705-023-00229-5 (PMC10039884; doi:10.1038/s43705-023-00229-5)
Supplement: Supplementary file 14 — Figure S14 [file 43705_2023_229_MOESM14_ESM.pdf]

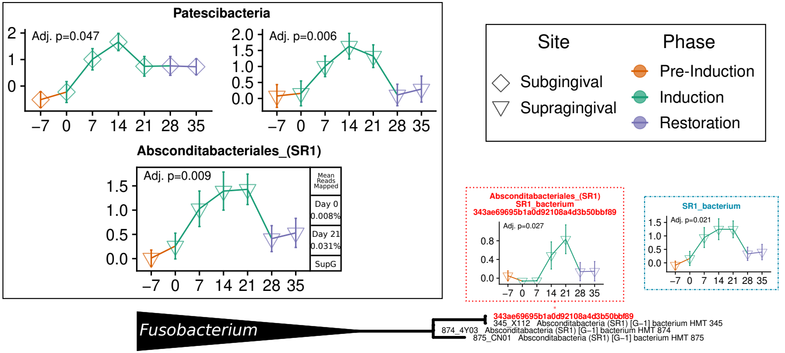

Patescibacteria
Adj. p=0.047
Adj. p=0.006
Absconditabacteriales_(SR1)
Adj. p=0.009
Mean Reads Mapped
Day 0 0.008%
Day 21 0.031%
SupG
Site
Subgingival
Supragingival
Phase
Pre-Induction
Induction
Restoration
Absconditabacteriales_(SR1)
SR1_bacterium
343ae69695b1a0d92108a4d3b50bbf89
Adj. p=0.027
SR1_bacterium
Adj. p=0.021
Fusobacterium
343ae69695b1a0d92108a4d3b50bbf89
345_X112 Absconditabacteria (SR1) [G−1] bacterium HMT 345
874_4Y03 Absconditabacteria (SR1) [G−1] bacterium HMT 874
875_CN01 Absconditabacteria (SR1) [G−1] bacterium HMT 875
