# Supplementary material for: Suspension of oral hygiene practices highlights key bacterial shifts in saliva, tongue, and tooth plaque during gingival inflammation and resolution
Source: ISME Commun. 2023 Mar 25;3:23. doi: 10.1038/s43705-023-00229-5 (PMC10039884; doi:10.1038/s43705-023-00229-5)
Supplement: Supplementary file 15 — Figure S15 [file 43705_2023_229_MOESM15_ESM.pdf]

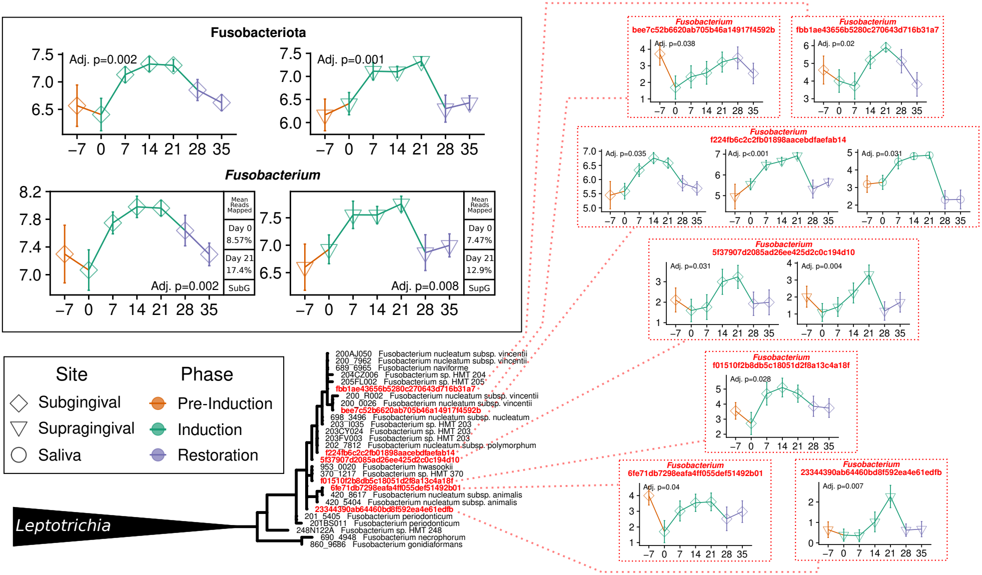

Fusobacteriota
Adj. p=0.002
Adj. p=0.001
Fusobacterium
Mean Reads Mapped
Day 0
8.57%
Day 21
17.4%
SubG
Adj. p=0.002
Mean Reads Mapped
Day 0
7.47%
Day 21
12.9%
SupG
Adj. p=0.008
Site
Subgingival
Supragingival
Saliva
Phase
Pre-Induction
Induction
Restoration
Leptotrichia
200AJ050 Fusobacterium nucleatum subsp. vincentii
200_7962 Fusobacterium nucleatum subsp. vincentii
689_6965 Fusobacterium naviforme
204CZ006 Fusobacterium sp. HMT 204
205FL002 Fusobacterium sp. HMT 205
fbb1ae43656b5280c270643d716b31a7
200_R002 Fusobacterium nucleatum subsp. vincentii
200_0026 Fusobacterium nucleatum subsp. vincentii
bee7c52b6620ab705b46a14917f4592b
698_3496 Fusobacterium nucleatum subsp. nucleatum
203_I035 Fusobacterium sp. HMT 203
203CY024 Fusobacterium sp. HMT 203
203FV003 Fusobacterium sp. HMT 203
202_7812 Fusobacterium nucleatum subsp. polymorphum
f224fb6c2c2fb01898aacebdfaefab14
5f37907d2085ad26ee425d2c0c194d10
953_0020 Fusobacterium hwasookii
370_1217 Fusobacterium sp. HMT 370
f01510f2b8db5c18051d2f8a13c4a18f
6fe71db7298eafa4ff055def51492b01
420_8617 Fusobacterium nucleatum subsp. animalis
420_5404 Fusobacterium nucleatum subsp. animalis
23344390ab64460bd8f592ea4e61edfb
201_5405 Fusobacterium periodonticum
201BS011 Fusobacterium periodonticum
248N122A Fusobacterium sp. HMT 248
690_4948 Fusobacterium necrophorum
860_9686 Fusobacterium gonidiaformans
Fusobacterium
bee7c52b6620ab705b46a14917f4592b
Adj. p=0.038
Fusobacterium
fbb1ae43656b5280c270643d716b31a7
Adj. p=0.02
Fusobacterium
f224fb6c2c2fb01898aacebdfaefab14
Adj. p=0.035
Adj. p<0.001
Adj. p=0.031
Fusobacterium
5f37907d2085ad26ee425d2c0c194d10
Adj. p=0.031
Adj. p=0.004
Fusobacterium
f01510f2b8db5c18051d2f8a13c4a18f
Adj. p=0.028
Fusobacterium
6fe71db7298eafa4ff055def51492b01
Adj. p=0.04
Fusobacterium
23344390ab64460bd8f592ea4e61edfb
Adj. p=0.007
