# Supplementary material for: Suspension of oral hygiene practices highlights key bacterial shifts in saliva, tongue, and tooth plaque during gingival inflammation and resolution
Source: ISME Commun. 2023 Mar 25;3:23. doi: 10.1038/s43705-023-00229-5 (PMC10039884; doi:10.1038/s43705-023-00229-5)
Supplement: Supplementary file 19 — Figure S19 [file 43705_2023_229_MOESM19_ESM.pdf]

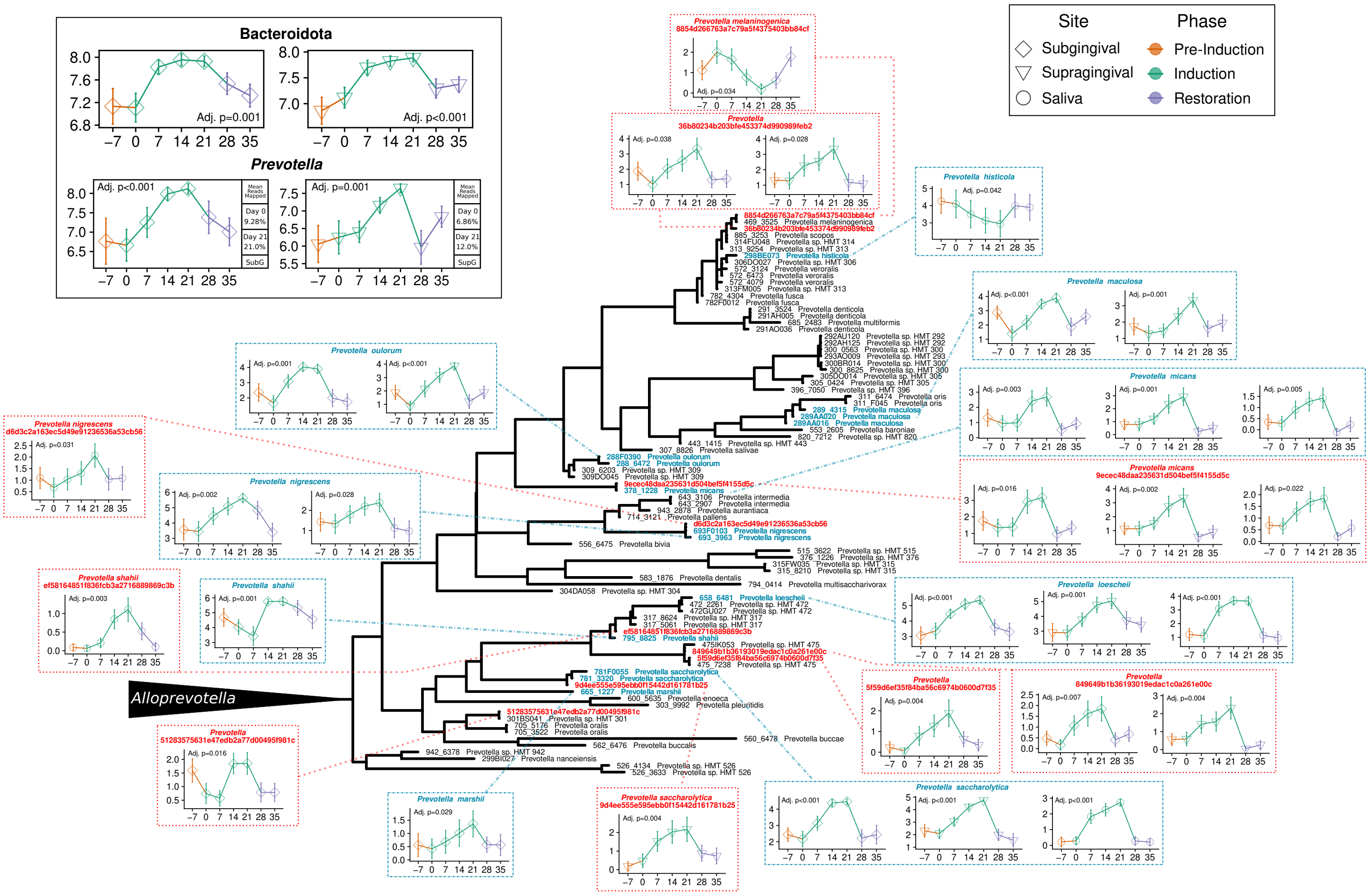

Site
Subgingival
Supragingival
Saliva
Phase
Pre-Induction
Induction
Restoration
Bacteroidota
Adj. p=0.001
Adj. p<0.001
Prevotella
Adj. p<0.001
Mean Reads Mapped
Day 0 9.28%
Day 21 21.0%
SubG
Adj. p=0.001
Mean Reads Mapped
Day 0 6.86%
Day 21 12.0%
SupG
Prevotella melaninogenica
8854d266763a7c79a5f4375403bb84cf
Adj. p=0.034
Prevotella
36b80234b203bfe453374d990989feb2
Adj. p=0.038
Adj. p=0.028
Prevotella histicola
Adj. p=0.042
Prevotella maculosa
Adj. p<0.001
Adj. p=0.001
Prevotella micans
Adj. p=0.003
Adj. p=0.001
Adj. p=0.005
Prevotella micans
9ecec48daa235631d504bef5f4155d5c
Adj. p=0.016
Adj. p=0.002
Adj. p=0.022
Prevotella oulorum
Adj. p=0.001
Adj. p<0.001
Prevotella nigrescens
d6d3c2a163ec5d49e91236536a53cb56
Adj. p=0.031
Prevotella nigrescens
Adj. p=0.002
Adj. p=0.028
Prevotella shahii
ef58164851f836fcb3a2716889869c3b
Adj. p=0.003
Prevotella shahii
Adj. p=0.001
Prevotella loescheii
Adj. p<0.001
Adj. p=0.001
Adj. p<0.001
Prevotella
5f59d6ef35f84ba56c6974b0600d7f35
Adj. p=0.004
Prevotella
849649b1b36193019edac1c0a261e00c
Adj. p=0.007
Adj. p=0.004
Prevotella
51283575631e47edb2a77d00495f981c
Adj. p=0.016
Prevotella marshii
Adj. p=0.029
Prevotella saccharolytica
9d4ee555e595ebb0f15442d161781b25
Adj. p=0.004
Prevotella saccharolytica
Adj. p<0.001
Adj. p<0.001
Adj. p<0.001
Alloprevotella
8854d266763a7c79a5f4375403bb84cf
469_3525 Prevotella melaninogenica
36b80234b203bfe453374d990989feb2
885_3253 Prevotella scopos
314FU048 Prevotella sp. HMT 314
313_9254 Prevotella sp. HMT 313
298BE073 Prevotella histicola
306DO027 Prevotella sp. HMT 306
572_3124 Prevotella veroralis
572_6473 Prevotella veroralis
572_4079 Prevotella veroralis
313FM005 Prevotella sp. HMT 313
782_4304 Prevotella fusca
782F0012 Prevotella fusca
291_3524 Prevotella denticola
291AH005 Prevotella denticola
685_2483 Prevotella multiformis
291AO036 Prevotella denticola
292AU120 Prevotella sp. HMT 292
292AH125 Prevotella sp. HMT 292
300_0563 Prevotella sp. HMT 300
293AO009 Prevotella sp. HMT 293
300BR014 Prevotella sp. HMT 300
300_8625 Prevotella sp. HMT 300
305DO014 Prevotella sp. HMT 305
305_0424 Prevotella sp. HMT 305
396_7050 Prevotella sp. HMT 396
311_6474 Prevotella oris
311_F045 Prevotella oris
289_4315 Prevotella maculosa
289AA020 Prevotella maculosa
289AA016 Prevotella maculosa
553_2605 Prevotella baroniae
820_7212 Prevotella sp. HMT 820
443_1415 Prevotella sp. HMT 443
307_8826 Prevotella salivae
288F0390 Prevotella oulorum
288_6472 Prevotella oulorum
309_6203 Prevotella sp. HMT 309
309DO045 Prevotella sp. HMT 309
9ecec48daa235631d504bef5f4155d5c
378_1228 Prevotella micans
643_3106 Prevotella intermedia
643_2907 Prevotella intermedia
943_2878 Prevotella aurantiaca
714_3121 Prevotella pallens
d6d3c2a163ec5d49e91236536a53cb56
693F0103 Prevotella nigrescens
693_3963 Prevotella nigrescens
556_6475 Prevotella bivia
515_3622 Prevotella sp. HMT 515
376_1226 Prevotella sp. HMT 376
315FW035 Prevotella sp. HMT 315
315_8210 Prevotella sp. HMT 315
583_1876 Prevotella dentalis
794_0414 Prevotella multisaccharivorax
304DA058 Prevotella sp. HMT 304
658_6481 Prevotella loescheii
472_2261 Prevotella sp. HMT 472
472GU027 Prevotella sp. HMT 472
317_8624 Prevotella sp. HMT 317
317_5061 Prevotella sp. HMT 317
ef58164851f836fcb3a2716889869c3b
795_8825 Prevotella shahii
475IK053 Prevotella sp. HMT 475
849649b1b36193019edac1c0a261e00c
5f59d6ef35f84ba56c6974b0600d7f35
475_7238 Prevotella sp. HMT 475
781F0055 Prevotella saccharolytica
781_3320 Prevotella saccharolytica
9d4ee555e595ebb0f15442d161781b25
665_1227 Prevotella marshii
600_5635 Prevotella enoeca
303_9992 Prevotella pleuritidis
51283575631e47edb2a77d00495f981c
301BS041 Prevotella sp. HMT 301
705_5176 Prevotella oralis
705_3522 Prevotella oralis
560_6478 Prevotella buccae
562_6476 Prevotella buccalis
942_6378 Prevotella sp. HMT 942
299BI027 Prevotella nanceiensis
526_4134 Prevotella sp. HMT 526
526_3633 Prevotella sp. HMT 526
