# Supplementary material for: Suspension of oral hygiene practices highlights key bacterial shifts in saliva, tongue, and tooth plaque during gingival inflammation and resolution
Source: ISME Commun. 2023 Mar 25;3:23. doi: 10.1038/s43705-023-00229-5 (PMC10039884; doi:10.1038/s43705-023-00229-5)
Supplement: Supplementary file 23 — Supplemental Information [file 43705_2023_229_MOESM23_ESM.docx]

### Supplementary Information

Interpreting compositional abundance shifts

The underlying abundance data in this study are gene counts,which are positive integer values that are often transformed prior to analysis and interpretation. Gene/taxon abundance data, when presented as raw counts or expressed as proportions of the sample, are compositional in nature (Gloor et al., 2017). There are significant and ongoing discussions about the impact this property has on the use and interpretation of community gene abundance data and the appropriate application of statistical methods (Quinn et al., 2019). The primary limitation to highlight for this work is the difficulty in interpreting the direction of change in proportional or percent abundance data. For example, as a percentage of the subgingival community, the genus *Streptococcus* decreased from 17.89% to 3.91%, while a simultaneous increase is observed in *Prevotella* which increased from 21.63% to 40.12%, on average. Without additional validation such as quantitative PCR analysis, there is an unresolvable ambiguity about whether the near-doubling of *Prevotella* is, in fact, a near-doubling of the number of *Prevotella* cells in the environment or whether a decline in other community members (such as *Streptococcus*) is leading to the appearance of an increase in *Prevotella* proportional abundances. In reality, it is likely some combination of those two possibilities and our observed abundances are the result of that push and pull.

In this study, two types of abundance data are routinely presented: proportional/percent abundances of gene counts and CLR-transformed gene counts. The former are generally more intuitive values that represent the proportion of a whole, while the latter transforms all counts as a log-ratio using the geometric mean of the sample as the denominator. While the CLR-transform benchmarks all taxon abundances relative to the average taxon in that sample and does not place the transformed values on a simplex as proportional/percentage values are,the interpretation of CLR-transformed values is less intuitive. The CLR-transformed abundances can be interpreted as the fold-difference in abundance between the scrutinized taxon and the average taxonomic unit at that level. A CLR-transformed value of 0 implies that the taxon has the same abundance as the average taxonomic unit (after the addition of pseudocounts). Positive values indicate a larger fold difference compared to the community average and negative values indicate taxonomic counts that are lower than the average taxon in the sample.

For these reasons stated above, statements about the absolute abundance of the organisms described in this study cannot be made with this data and to do so would require additional laboratory analysis. However, the pre-EGM and recovery phase samples represent important benchmarks that enable comparisons of the overall importance of each taxonomic unit relative to the average taxon in the community. When these benchmarks are compared to the weekly time points collected during the induction phase, the temporal trajectory of each taxon after suspension of OHP, in terms of its relative weight in the community, can be stated.

**Phylogenetic and taxonomic resolution**

ASVs within the blue (*Actinomcyes*) and yellow (*Streptococcus*) complexes did not frequently classify down to species level with the naïve Bayes algorithm used. The phylogenetic trees (Figures S6 and S8) help visualize why: clades within these groups often have multiple species designations with very little nucleotide variation between them. This results in an unacceptably low confidence score as it fails to differentiate between species with highly similar 16S gene regions, and the classification process defaults to a genus level annotation. Nevertheless, the phylogenetic reference trees help situate these ASVs relative to reference sequences and one another, and helps increase the resolution for those taxa. Future studies probing these microbial groups with DNA sequencing should consider a gene fragment or gene with higher within-group nucleotide diversity to clarify the uncertain phylogenies and classifications provided here and in studies with similar sequencing protocols.

Descriptions of bacterial dynamics across phyla

**Spirochaetota**

Supragingival and subgingival plaque samples showed significant increases in CLR-transformed abundances of reads classified to the phylum Spirochatota over the gingivitis induction period (Figure 4). The vast

majority of reads classified to this phylum were further classified to the genus *Treponema*, which showed the same significantly increasing trends in supra- and subgingival plaque samples (Figure 5). At the species level, six species demonstrated significant trends over gingivitis induction, with subgingival levels of *T. medium*, *T. socranskii*, and *T. maltophilum* almost doubling in mean relative abundance. The lower abundance species T. vincenitniicreased nearly 6-fold from 0.03% to 0.19% mean relative abundance in the subgingival plaque, and doubled its mean abundance in supragingival plaque from 0.03% to 0.06%. The two remaining significant species, *T. genomosp*. and *T. refringens*, were ultra-low abundance (<0.01%) but nonetheless saw a significant decrease in the supragingival and subgingival plaque, respectively (Figure S1, Table S1). At the ASV level, three variants increased over the induction period: one *T. vincentii* and one *T. medium* ASV increased in subgingival plaque, and one *T. socranskii* ASV increased in the supragingival plaque (Figure S1).

**Pseudomonadota**

The phylum Pseudomonadota decreased sharply and significantly in the tooth plaque samples after the first week of induction (Figure 4), driven by the most abundant genus *Neisseria* (Figure 5), which accounted for 9.58% and 12.8% on day 0 in the subgingival and supragingival plaque, respectively, and fell significantly to

2.23% and 4.96% by day 21. Interestingly, *Neisseria* increases in abundance for the first two weeks after suspension of OHP before rapidly falling for an overall decrease in community representation. An ASV classifying to *Kingella*, a close neighbour of *Neisseria*, decreased significantly in supragingival plaque samples over the induction period (Figure S4).

In the subgingival plaque, *Haemophilus* showed a 30-fold decrease over the gingivitis induction period, from 3.41% to 0.11% mean read abundance and a 5-fold decrease from 3.55% to 0.63% in the supragingival plaque. Three ASVs that classified to *Haemophilus* and all placed closely on the reference tree to *H. parainfluenzae* had significantly decreasing trends in the subgingival plaque, with one of the ASVs also decreasing significantly in the supragingival plaque (Figure S2). Similarly, sequences from the genus *Lautropia* decreased 3-fold in both supra- and subgingival plaque, though the result was only statistically significant for the supragingival plaque (Figures 5, S3).

In contrast with the generally decreasing trends in this phylum, the genus *Aggregatibacter* demonstrated an increasing trend in both tooth plaques and on the tongue. This genus was the sole taxonomic unit (whether phylum, genus, species, or ASV) that significantly trended in either direction on the tongue. In both tooth plaque environments, *Aggregatibacter* almost doubled their relative abundance from about 0.8% to 1.5%, but increased almost 7-fold on the tongue from 0.014% to 0.093% (Figure S2).

Located between *Aggregatibacter* and *Neisseria* on the reference tree, three additional ASVs increased significantly over the induction phase. In aggregate, *Cardiobacterium valvarum* sequences increased significantly in both tooth plaque environments, while a specific *C. valvarum* ASV increased significantly only in the supragingival plaque samples. An ASV that classified as *Comamonadaceae* and placed on the reference tree near genus *Ottowia* increased in the supragingival plaque. The final significant ASV in this group classified to *Propionivibrio*, placed near genus *Rhodocyclus*, and increased in the subgingival plaque (Figure S3).

**Actinomycetota**

Abundances of Actinomycetota fell quickly and significantly after OHP were suspended (Figure 4, Table S1). This was driven by a significant 4.2 and 3.8-fold decrease in abundance of the genus *Actinomcyes* in the subgingival and supragingival plaque, respectively (Figure 5, Table S1). Three ASVs in this genus significantly decreased in tooth plaque samples, including two that placed on the reference tree near a group of sequences with

low nucleotide variation in the sequenced V4-V5 region that originated from *A. oris*, *A. naeslundii*, *A. viscosus*, and

*A. johnsonii*. The third ASV placed most closely to *A. gerencseriae* and decreased significantly in the subgingival plaque.

The second-most abundant genus in the phylum was *Rothia*,which decreased in both the subgingival and supragingival plaque, seeing a significant 20.8 and 8.8-fold decrease, respectively. In the subgingival plaque, *Rothia* mean abundance fell from 1.24% to 0.06%, with supragingival plaque mean abundance similarly dropping from 1.62% to 0.18%. Two ASVs that classified to *Rothia* significantly decreased over the induction phase. The first decreased significantly in the subgingival plaque and placed on the reference tree most closely to *R. aeria*. The second decreased significantly in both plaque types and placed most closely to *R. dentocariosa*.

A low-abundance uncultivated group F0332 in the family *Actinomycetaceae* decreased significantly in the supragingival plaque from 0.36% to 0.07%, with a concurrent 8-fold, but not statistically significant, decrease in the subgingival plaque. This was driven by an ASV that placed with sequences belonging to genus *Peptidiphaga* When sequence abundances were aggregated at the genus level, *Corynebacterium* abundance increased significantly only in the saliva, driven by sequences that classified down to *C. matruchotii*. More in line with other members of its phylum, *C. durum* decreased in abundance in both tooth plaque sets, led by one primary ASV (Figure S5, Table S1). (Figure S6, Table S1). One final very low abundance ASV that placed near members of the *Arachnia* genus increased significantly in the fourth week of induction (Figure S5).

**Bacillota**

The phylum Bacillota is a large and diverse group,so while it decreased significantly in abundance in the subgingival plaque after the first full week of the cessation of OHP (Figure 4), it also contains several lower abundance members that increased significantly in abundance over the same period. The phylum-level decrease following the first week was driven primarily by a reduction ofthe most abundant genus, *Streptococcus*, and to a lesser extent *Granulicatella*. On the other hand, *Selenomonas*, *Dialister*, *Johnsonella*, *Gemella*, *Parvimonas*, *Catonella*, *Centipeda*, and *Abiotrophia* are all low abundance genera that saw significant increases over gingivitis induction (Figure 3, Table S1). Bacillota also contains one of the most abundant genera that did not significantly

increase or decrease in any site, *Veillonella*.

Over the induction period, *Granulicatella* saw its relative proportion in the community shrink by a factor of 4 in both the tooth plaque environments. Meanwhile, neighbouring genus *Abiotrophia*, increased its representation in saliva samples by 4-fold, and *Gemella* increased significantly in the supragingival plaque but began declining by week 4 of suspended OHP (Figure S7). One ASV drove the *Granulicatella* decreases, most closely placing on the reference tree near *G. adiacens*, while another ASV drove the *Abiotrophia* increase in saliva, placing close to an *A. defectiva* reference sequence (Figure S7).

Members of the genus *Streptococcus* saw a steady decrease after suspension of OHP in both tooth plaque environments, with a 4.6-fold decrease in subgingival plaque and 2.9-fold decrease in supragingival plaque by day

21. Three streptococcal ASVs decreased significantly in subgingival plaque, falling on the reference tree near *S. oralis*/*S. mitis*, *S. parasanguinis*/*S. australis*, and *S. salivarius*/*S. vestibularis*/*S. thermophilus*. A final ASV, high in abundance and placing near *S. sanguinis*, decreased significantly in both the subgingival and supragingival plaques (Figure S8).

The genus *Selenomonas* increased significantly in the tooth plaque, increasing from 1.9% of subgingival samples at day 0 to 3.1% at day 21. The closely related and lower abundance genus *Centipeda* also increased in the tooth plaque samples over the induction phase. Two of the significant *Selenomonas* ASVs increased significantly in saliva samples, placing near *S. infelix* and *S. artemidis*. At the species and ASV level, *S. sputigena* sequences increased significantly in the tooth plaque samples and accounted for nearly half of *Selenomonas* sequences in subgingival plaque at day 21 (Figure S10, Table S1).

Additional low abundance members of the Bacillota phylum include *Peptococcus* and *Dialister* (Figure S9), *Catonella*, *Johnsonella*, *Lachnoanaerobaculum*, and Clostridia UCG-014 (Figure S11), and *Parvimonas* (Figure S12), which were generally found to be increasing in the tooth plaque samples, particularly subgingival samples.

**Patescibacteria & *Campylobacter*ota**

Patescibacteria, sometimes known as SR1, is a very low abundance group that showed significant increases in the tooth plaque samples, with the *Absconditabacteriales* group increasing from 0.008% of reads mapped to 0.031% by day 21 in subgingival samples. One ASV in this group significantly increased in supragingival samples, and mapped most closely to HMT 345 in the eHOMD reference database (Figure S14). A genus-level designation JGI 0000069-P22 increased significantly in both tooth plaque samples, a result that was significant only in supragingival plaque at the species level as *Gracilibacteria* bacterium, and for an ASV that placed near eHOMD HMT 872 (Figure S13).

The phylum *Campylobacter*ota increased significantly in the tooth plaque samples. At the species level, *C. showae* sequences increased significantly in the supragingival plaque (Figure S13).

**Fusobacterota**

As a phylum, the Fusobacterota doubled their mean reads mapped from day 0 to day 21 in both tooth plaque environments. This was driven by members of the genus *Fusobacterium*, which doubled from 8.6% to 17.4% in the subgingival plaque, and the genus *Leptotrichia*, which doubled from 2.0% to 4.1% in subgingival plaque.

*Fusobacterium* sequences included 7 ASVs that increased in the tooth plaque, and one that increased in the tooth plaque and saliva samples. The ASV that increased significantly in all three environments placed most closely to *F. nucleatum* subsp. *polymorphum* (HMT 203). Tooth plaque ASVs placed near *F. nucleatum* subsp. *vincentii* (HMT 200, 205), *F. hwasookii* (HMT 370, 953), and *F. nucleatum* subsp. animalis (HMT 420) (Figure S15).

At the species level, *Leptotrichia* saw significant increases in reads mapping to *L. buccalis*, *L. shahii*, and a significant decrease in supragingival plaque in low abundance species-level designation *Leptotrichia*-like sp. (Figure S16). The *L. buccalis* increase was driven by an ASV that mapped near HMT 563, and similarly the *L. shahii* with HMT 214. Additional *Leptotrichia* ASVs that placed near HMT 219 and HMT 417 increased significantly in the subgingival plaque (Figure S16).

**Bacteroidota**

The Bacteroidota are another large phylum with a diverse set of dynamics exhibited after the suspension of OHP. As a phylum, its members nearly double their community proportions by day 21, accounting for a mean of 40% of subgingival samples at this time point. More than half of this abundance belongs to the genus *Prevotella* which increased significantly in both tooth plaque environments. The genera *Porphyromonas*, *Alloprevotella*, *Tannerella*, and genus-level designation F0058 also increased significantly in the tooth plaque samples.

The genus *Prevotella* accounted for an average of 8.9% of reads mapped at day 0 and increased to 21.0% by day 21 in the subgingival plaque with a similar increase observed in the supragingival plaque. At the species level, a wide number of named *Prevotella* species increased in one or both of the tooth plaque environments and saliva samples: *P. shahii*, *P. nigrescens*, *P. loescheii*, *P. saccharolytica*, *P. micans*, *P. maculosa*, *P. oulorum*, and *P. marshii*, while *P. histicola* sequences saw their salivary proportion decrease significantly from 1.9% at day 0 to 0.7% at day 21.

The ASV-level results reveal the same diversity seen in the species-level aggregations. In total 9 ASVs classifying to *Prevotella* increased significantly in the tooth plaque and/or saliva. Three of these ASVs placed near the significant species-level designations *P. micans*, *P. nigrescens*, *P. shahii*, and *P. saccharolytica*. Of the remaining 5 ASVs, two placed near *P. melaninogenica*, and eHOMD reference sequences HMT 475 and HMT 301. The two

*P. melaninogenica* ASVs include one that decreases significantly over time and another that increases, in a rare example of opposing dynamics in such closely related units (Figure S19).

The second-most abundant genus in the Bacteroidota was *Capnocytophaga*, which saw non-significant increases in abundance across all sample types. The only named species with a significant increase was *C. granulosa*, which increased significantly in supragingival plaque. Counter to the dynamics in the genus, *C. gingivalis* and *C. leadbetteri* sequences decreased significantly in subgingival plaque, though this followed a period of initial increase after the suspension of OHP. Nearby genus *Bergeyella* had species-level designation “uncultured *Bergeyella*” increase in both tooth plaque types and saliva samples (Figure S17).

Members of the genus *Porphyromonas* doubled their proportion of the tooth plaque community, on average, over the induction phase. A similar increase was seen in the saliva but was not statistically significant after p-value correction. At the species level, *P. catoniae* significantly increased over two-fold in the subgingival plaque and over 5-fold in supragingival plaque. One ASV matched the dynamics of the species-level aggregate for *P. catoniae* and increased significant in both tooth plaques and saliva. A second significant *Porphyromonas* ASV increased from low abundance after suspension of OHP in supragingival plaque and saliva, placing near eHOMD HMT 275, HMT 277, and HMT 284 (Figure S18).

*Alloprevotella* increase their proportion of the community nearly 5-fold in the subgingival plaque, from 0.8% to 4.0%, with a significant two-fold increase in supragingival plaque also observed. The species *A. tannerae* increased its proportion over 8-fold between day 0 and day 21 in the subgingival plaque. Two ASVs placed near *A. tannerae* (HMT 466) sequences and increased significantly in the subgingival plaque (Figure S18).

As a genus, *Tannerella* sequences increased roughly 3-fold in both tooth plaque environments. *T. forsythia*

proportions decreased significantly in the supragingival plaque samples, from a mean of 0.05% to zero detection

across all subject samples. Three *Tannerella* ASVs increased significantly in both tooth plaque environments, placing closer to HMT 286, HMT 916, and HMT 808 than the *T. forsythia* reference HMT 613 (Figure S18).

The genus-level designation F0058, belonging to the family *Paludibacteraceae*, increased significantly in both tooth plaque samples. While no one ASV in this group met the significance threshold, representative sequences from this taxonomic group placed near *Bacteroidales* G-2 bacterium (HMT 274). Unlike its phylogenetic neighbours, the low-abundance genus Bacteroides decreased significantly in both tooth plaque environments over the induction period (Figure S18).

Bibliography

Gloor, G. B., Macklaim, J. M., Pawlowsky-Glahn, V., and Egozcue, J. J. *Microbiome datasets are compositional: and this is not optional*. *Frontiers in Microbiology*, 8:2224, 2017.

Quinn, T. P., Erb, I., Gloor, G., Notredame, C., Richardson, M. F., and Crowley, T. M. *A field guide for the compositional analysis of any-omics data*. *GigaScience*, 8(9):giz107, 2019.

Figure S1. ***Treponema***

Sequence abundances aggregated at the phylum and genus level (solid black outline), species level (blue dashed outline) and ASV level (red dotted outline). Y-axis is CLR-transformed reads and x-axis is days. Phylogenetic tree situates differentially abundant named species (blue) and ASVs (red) alongside the eHOMD reference sequences (black). p-values are the result of testing the null hypothesis that the slope is zero over the induction phase, with only significant results shown.

Figure S2. ***Haemophilus*, *Aggregatibacter***

Sequence abundances aggregated at the phylum and genus level (solid black outline), species level (blue dashed outline) and ASV level (red dotted outline). Y-axis is CLR-transformed reads and x-axis is days. Phylogenetic tree situates differentially abundant named species (blue) and ASVs (red) alongside the eHOMD reference sequences (black). p-values are the result of testing the null hypothesis that the slope is zero over the induction phase, with only significant results shown.

Figure S3. ***Lautropia*, *Cardiobacterium***

Sequence abundances aggregated at the phylum and genus level (solid black outline), species level (blue dashed outline) and ASV level (red dotted outline). Y-axis is CLR-transformed reads and x-axis is days. Phylogenetic tree situates differentially abundant named species (blue) and ASVs (red) alongside the eHOMD reference sequences (black). p-values are the result of testing the null hypothesis that the slope is zero over the induction phase, with only significant results shown.

Figure S4. ***Neisseria*, *Kingella***

Sequence abundances aggregated at the phylum and genus level (solid black outline), species level (blue dashed outline) and ASV level (red dotted outline). Y-axis is CLR-transformed reads and x-axis is days. Phylogenetic tree situates differentially abundant named species (blue) and ASVs (red) alongside the eHOMD reference sequences (black). p-values are the result of testing the null hypothesis that the slope is zero over the induction phase, with only significant results shown.

Figure S5. ***Corynebacterium***

Sequence abundances aggregated at the phylum and genus level (solid black outline), species level (blue dashed outline) and ASV level (red dotted outline). Y-axis is CLR-transformed reads and x-axis is days. Phylogenetic tree situates differentially abundant named species (blue) and ASVs (red) alongside the eHOMD reference sequences (black). p-values are the result of testing the null hypothesis that the slope is zero over the induction phase, with only significant results shown.

Figure S6. ***Actinomyces*, *Rothia***

Sequence abundances aggregated at the phylum and genus level (solid black outline), species level (blue dashed outline) and ASV level (red dotted outline). Y-axis is CLR-transformed reads and x-axis is days. Phylogenetic tree situates differentially abundant named species (blue) and ASVs (red) alongside the eHOMD reference sequences (black). p-values are the result of testing the null hypothesis that the slope is zero over the induction phase, with only significant results shown.

Figure S7. ***Granulicatella*, *Abiotrophia*, *Gemella***

Sequence abundances aggregated at the phylum and genus level (solid black outline), species level (blue dashed outline) and ASV level (red dotted outline). Y-axis is CLR-transformed reads and x-axis is days. Phylogenetic tree situates differentially abundant named species (blue) and ASVs (red) alongside the eHOMD reference sequences (black). p-values are the result of testing the null hypothesis that the slope is zero over the induction phase, with only significant results shown.

Figure S8. ***Streptococcus***

Sequence abundances aggregated at the phylum and genus level (solid black outline), species level (blue dashed outline) and ASV level (red dotted outline). Y-axis is CLR-transformed reads and x-axis is days. Phylogenetic tree situates differentially abundant named species (blue) and ASVs (red) alongside the eHOMD reference sequences (black). p-values are the result of testing the null hypothesis that the slope is zero over the induction phase, with only significant results shown.

Figure S9. ***Veillonella*, *Dialister*, *Peptococcus***

Sequence abundances aggregated at the phylum and genus level (solid black outline), species level (blue dashed outline) and ASV level (red dotted outline). Y-axis is CLR-transformed reads and x-axis is days. Phylogenetic tree situates differentially abundant named species (blue) and ASVs (red) alongside the eHOMD reference sequences (black). p-values are the result of testing the null hypothesis that the slope is zero over the induction phase, with only significant results shown.

Figure S10. ***Selenomonas*, *Centipeda***

Sequence abundances aggregated at the phylum and genus level (solid black outline), species level (blue dashed outline) and ASV level (red dotted outline). Y-axis is CLR-transformed reads and x-axis is days. Phylogenetic tree situates differentially abundant named species (blue) and ASVs (red) alongside the eHOMD reference sequences (black). p-values are the result of testing the null hypothesis that the slope is zero over the induction phase, with only significant results shown.

Figure S11. ***Catonella*, *Johnsonella*, *Oribacterium***

Sequence abundances aggregated at the phylum and genus level (solid black outline), species level (blue dashed outline) and ASV level (red dotted outline). Y-axis is CLR-transformed reads and x-axis is days. Phylogenetic tree situates differentially abundant named species (blue) and ASVs (red) alongside the eHOMD reference sequences (black). p-values are the result of testing the null hypothesis that the slope is zero over the induction phase, with only significant results shown.

Figure S12. ***Parvimonas***

Sequence abundances aggregated at the phylum and genus level (solid black outline), species level (blue dashed outline) and ASV level (red dotted outline). Y-axis is CLR-transformed reads and x-axis is days. Phylogenetic tree situates differentially abundant named species (blue) and ASVs (red) alongside the eHOMD reference sequences (black). p-values are the result of testing the null hypothesis that the slope is zero over the induction phase, with only significant results shown.

Figure S13. ***Campylobacter*, *Gracilibacteria* (GN02, BD1-5, or SN-2)**

Sequence abundances aggregated at the phylum and genus level (solid black outline), species level (blue dashed outline) and ASV level (red dotted outline). Y-axis is CLR-transformed reads and x-axis is days. Phylogenetic tree situates differentially abundant named species (blue) and ASVs (red) alongside the eHOMD reference sequences (black). p-values are the result of testing the null hypothesis that the slope is zero over the induction phase, with only significant results shown.

Figure S14. ***Absconditabacteriales***

Sequence abundances aggregated at the phylum and genus level (solid black outline), species level (blue dashed outline) and ASV level (red dotted outline). Y-axis is CLR-transformed reads and x-axis is days. Phylogenetic tree situates differentially abundant named species (blue) and ASVs (red) alongside the eHOMD reference sequences (black). p-values are the result of testing the null hypothesis that the slope is zero over the induction phase, with only significant results shown.

Figure S15. ***Fusobacteria***

Sequence abundances aggregated at the phylum and genus level (solid black outline), species level (blue dashed outline) and ASV level (red dotted outline). Y-axis is CLR-transformed reads and x-axis is days. Phylogenetic tree situates differentially abundant named species (blue) and ASVs (red) alongside the eHOMD reference sequences (black). p-values are the result of testing the null hypothesis that the slope is zero over the induction phase, with only significant results shown.

Figure S16. ***Leptotrichia***

Sequence abundances aggregated at the phylum and genus level (solid black outline), species level (blue dashed outline) and ASV level (red dotted outline). Y-axis is CLR-transformed reads and x-axis is days. Phylogenetic tree situates differentially abundant named species (blue) and ASVs (red) alongside the eHOMD reference sequences (black). p-values are the result of testing the null hypothesis that the slope is zero over the induction phase, with only significant results shown.

Figure S17. ***Capnocytophaga*, *Bergeyella***

Sequence abundances aggregated at the phylum and genus level (solid black outline), species level (blue dashed outline) and ASV level (red dotted outline). Y-axis is CLR-transformed reads and x-axis is days. Phylogenetic tree situates differentially abundant named species (blue) and ASVs (red) alongside the eHOMD reference sequences (black). p-values are the result of testing the null hypothesis that the slope is zero over the induction phase, with only significant results shown.

Figure S18. ***Alloprevotella*, *Tannerella*, *Porphyromonas***

Sequence abundances aggregated at the phylum and genus level (solid black outline), species level (blue dashed outline) and ASV level (red dotted outline). Y-axis is CLR-transformed reads and x-axis is days. Phylogenetic tree situates differentially abundant named species (blue) and ASVs (red) alongside the eHOMD reference sequences (black). p-values are the result of testing the null hypothesis that the slope is zero over the induction phase, with only significant results shown.

Figure S19. ***Prevotella***

Sequence abundances aggregated at the phylum and genus level (solid black outline), species level (blue dashed outline) and ASV level (red dotted outline). Y-axis is CLR-transformed reads and x-axis is days. Phylogenetic tree situates differentially abundant named species (blue) and ASVs (red) alongside the eHOMD reference sequences (black). p-values are the result of testing the null hypothesis that the slope is zero over the induction phase, with only significant results shown.

Figure S20. Genera with abundances that demonstrated positive slopes over the gingivitis induction period. Sampling time points are on the x-axis and CLR-transformed abundance values are on the y-axis. Mean abundance values for day 0 and day 21 are provided for reference. P-values are FDR-adjusted for multiple hypothesis test correction and results with p ≤0.05 considered significant.

Figure S21. Beta diversity boxplots showing intra- and inter-personal variation in microbial diversity. Boxplots showing the intra-subject and intra-time point Bray-Curtis dissimilarties between samples. Boxplots show the median, 1st, and 3rd quartiles and 1.5 times the inter-quartile range.
