# Supplementary material for: Suspension of oral hygiene practices highlights key bacterial shifts in saliva, tongue, and tooth plaque during gingival inflammation and resolution
Source: ISME Commun. 2023 Mar 25;3:23. doi: 10.1038/s43705-023-00229-5 (PMC10039884; doi:10.1038/s43705-023-00229-5)
Supplement: Supplementary file 11 — Figure S11 [file 43705_2023_229_MOESM11_ESM.pdf]

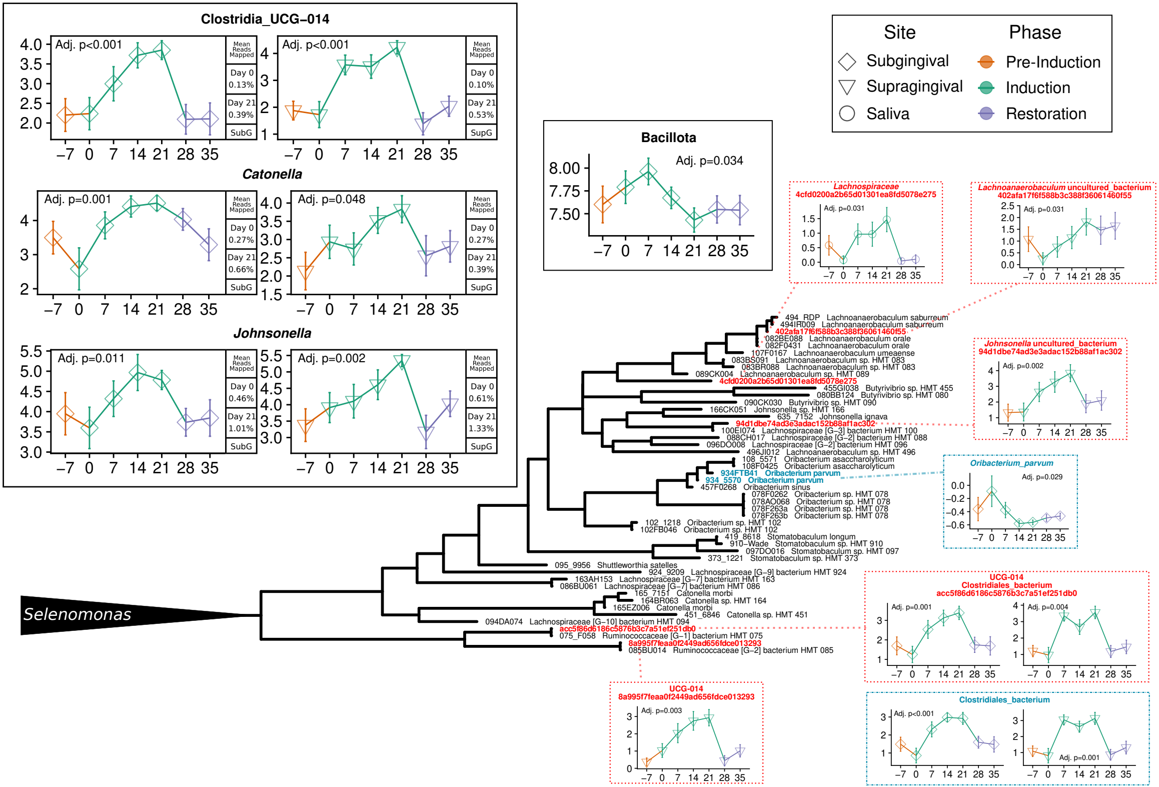

Clostridia_UCG-014
Adj. p<0.001
Mean Reads Mapped
Day 0 0.13%
Day 21 0.39%
SubG
Adj. p<0.001
Day 0 0.10%
Day 21 0.53%
SupG
Catonella
Adj. p=0.001
Day 0 0.27%
Day 21 0.66%
SubG
Adj. p=0.048
Day 0 0.27%
Day 21 0.39%
SupG
Johnsonella
Adj. p=0.011
Day 0 0.46%
Day 21 1.01%
SubG
Adj. p=0.002
Day 0 0.61%
Day 21 1.33%
SupG
Bacillota
Adj. p=0.034
Site
Subgingival
Supragingival
Saliva
Phase
Pre-Induction
Induction
Restoration
Lachnospiraceae
4cfd0200a2b65d01301ea8fd5078e275
Adj. p=0.031
Lachnoanaerobaculum uncultured_bacterium
402afa17f6f588b3c388f36061460f55
Adj. p=0.031
494_RDP Lachnoanaerobaculum saburreum
494IR009 Lachnoanaerobaculum saburreum
402afa17f6f588b3c388f36061460f55
082BE088 Lachnoanaerobaculum orale
082F0431 Lachnoanaerobaculum orale
107F0167 Lachnoanaerobaculum umeaense
083BS091 Lachnoanaerobaculum sp. HMT 083
083BR088 Lachnoanaerobaculum sp. HMT 083
089CK004 Lachnoanaerobaculum sp. HMT 089
4cfd0200a2b65d01301ea8fd5078e275
455GI038 Butyrivibrio sp. HMT 455
080BB124 Butyrivibrio sp. HMT 080
090CK030 Butyrivibrio sp. HMT 090
166CK051 Johnsonella sp. HMT 166
635_7152 Johnsonella ignava
94d1dbe74ad3e3adac152b88af1ac302
100EI074 Lachnospiraceae [G-3] bacterium HMT 100
088CH017 Lachnospiraceae [G-2] bacterium HMT 088
096DO008 Lachnospiraceae [G-2] bacterium HMT 096
496JI012 Lachnoanaerobaculum sp. HMT 496
108_5571 Oribacterium asaccharolyticum
108F0425 Oribacterium asaccharolyticum
934FTB41 Oribacterium parvum
934_5570 Oribacterium parvum
457F0268 Oribacterium sinus
078F0262 Oribacterium sp. HMT 078
078AO068 Oribacterium sp. HMT 078
078F263a Oribacterium sp. HMT 078
078F263b Oribacterium sp. HMT 078
102_1218 Oribacterium sp. HMT 102
102FB046 Oribacterium sp. HMT 102
419_8618 Stomatobaculum longum
910-Wade Stomatobaculum sp. HMT 910
097DO016 Stomatobaculum sp. HMT 097
373_1221 Stomatobaculum sp. HMT 373
095_9956 Shuttleworthia satelles
924_9209 Lachnospiraceae [G-9] bacterium HMT 924
163AH153 Lachnospiraceae [G-7] bacterium HMT 163
086BU061 Lachnospiraceae [G-7] bacterium HMT 086
165_7151 Catonella morbi
164BR063 Catonella sp. HMT 164
165EZ006 Catonella morbi
451_6846 Catonella sp. HMT 451
094DA074 Lachnospiraceae [G-10] bacterium HMT 094
acc5f86d6186c5876b3c7a51ef251db0
075_F058 Ruminococcaceae [G-1] bacterium HMT 075
8a995f7feaa0f2449ad656fdce013293
085BU014 Ruminococcaceae [G-2] bacterium HMT 085
Selenomonas
Johnsonella uncultured_bacterium
94d1dbe74ad3e3adac152b88af1ac302
Adj. p=0.002
Oribacterium_parvum
Adj. p=0.029
UCG-014
Clostridiales_bacterium
acc5f86d6186c5876b3c7a51ef251db0
Adj. p=0.001
Adj. p=0.004
UCG-014
8a995f7feaa0f2449ad656fdce013293
Adj. p=0.003
Clostridiales_bacterium
Adj. p<0.001
Adj. p=0.001
