# Supplementary material for: Suspension of oral hygiene practices highlights key bacterial shifts in saliva, tongue, and tooth plaque during gingival inflammation and resolution
Source: ISME Commun. 2023 Mar 25;3:23. doi: 10.1038/s43705-023-00229-5 (PMC10039884; doi:10.1038/s43705-023-00229-5)
Supplement: Supplementary file 12 — Figure S12 [file 43705_2023_229_MOESM12_ESM.pdf]

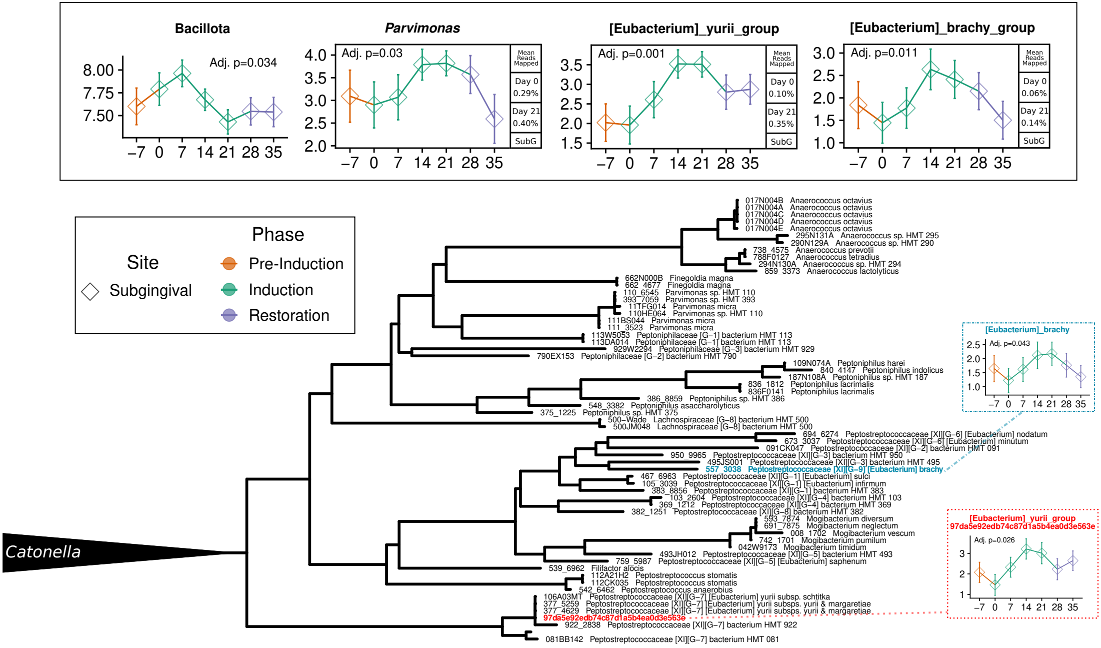

Bacillota
Adj. p=0.034
8.00
7.75
7.50
−7 0 7 14 21 28 35
Parvimonas
Adj. p=0.03
4.0
3.5
3.0
2.5
2.0
Mean Reads Mapped
Day 0 0.29%
Day 21 0.40%
SubG
[Eubacterium]_yurii_group
Adj. p=0.001
Day 0 0.10%
Day 21 0.35%
[Eubacterium]_brachy_group
Adj. p=0.011
Day 0 0.06%
Day 21 0.14%
Phase
Site
Subgingival
Pre-Induction
Induction
Restoration
017N004B Anaerococcus octavius
017N004A Anaerococcus octavius
017N004C Anaerococcus octavius
017N004D Anaerococcus octavius
017N004E Anaerococcus octavius
295N131A Anaerococcus sp. HMT 295
290N129A Anaerococcus sp. HMT 290
738_4575 Anaerococcus prevotii
788F0127 Anaerococcus tetradius
294N130A Anaerococcus sp. HMT 294
859_3373 Anaerococcus lactolyticus
662N000B Finegoldia magna
662_4677 Finegoldia magna
110_6545 Parvimonas sp. HMT 110
393_7059 Parvimonas sp. HMT 393
111FG014 Parvimonas micra
110HE064 Parvimonas sp. HMT 110
111BS044 Parvimonas micra
111_3523 Parvimonas micra
113W5053 Peptoniphilaceae [G−1] bacterium HMT 113
113DA014 Peptoniphilaceae [G−1] bacterium HMT 113
929W2294 Peptoniphilaceae [G−3] bacterium HMT 929
790EX153 Peptoniphilaceae [G−2] bacterium HMT 790
109N074A Peptoniphilus harei
840_4147 Peptoniphilus indolicus
187N108A Peptoniphilus sp. HMT 187
836_1812 Peptoniphilus lacrimalis
836F0141 Peptoniphilus lacrimalis
386_8859 Peptoniphilus sp. HMT 386
548_3382 Peptoniphilus asaccharolyticus
375_1225 Peptoniphilus sp. HMT 375
500−Wade Lachnospiraceae [G−8] bacterium HMT 500
500JM048 Lachnospiraceae [G−8] bacterium HMT 500
694_6274 Peptostreptococcaceae [XI][G−6] [Eubacterium] nodatum
673_3037 Peptostreptococcaceae [XI][G−6] [Eubacterium] minutum
091CK047 Peptostreptococcaceae [XI][G−2] bacterium HMT 091
950_9965 Peptostreptococcaceae [XI][G−3] bacterium HMT 950
495JS001 Peptostreptococcaceae [XI][G−3] bacterium HMT 495
557_3038 Peptostreptococcaceae [XI][G−9] [Eubacterium] brachy
467_6963 Peptostreptococcaceae [XI][G−1] [Eubacterium] sulci
105_3039 Peptostreptococcaceae [XI][G−1] [Eubacterium] infirmum
383_8856 Peptostreptococcaceae [XI][G−1] bacterium HMT 383
103_2604 Peptostreptococcaceae [XI][G−4] bacterium HMT 103
369_1212 Peptostreptococcaceae [XI][G−4] bacterium HMT 369
382_1251 Peptostreptococcaceae [XI][G−8] bacterium HMT 382
593_7874 Mogibacterium diversum
691_7875 Mogibacterium neglectum
008_1702 Mogibacterium vescum
742_1701 Mogibacterium pumilum
042W9173 Mogibacterium timidum
493JH012 Peptostreptococcaceae [XI][G−5] bacterium HMT 493
759_5987 Peptostreptococcaceae [XI][G−5] [Eubacterium] saphenum
539_6962 Filifactor alocis
112A21H2 Peptostreptococcus stomatis
112CK035 Peptostreptococcus stomatis
542_6462 Peptostreptococcus anaerobius
106A03MT Peptostreptococcaceae [XI][G−7] [Eubacterium] yurii subsp. schtitka
377_5259 Peptostreptococcaceae [XI][G−7] [Eubacterium] yurii subsps. yurii & margaretiae
377_4629 Peptostreptococcaceae [XI][G−7] [Eubacterium] yurii subsps. yurii & margaretiae
97da5e92edb74c87d1a5b4ea0d3e563e
922_2838 Peptostreptococcaceae [XI][G−7] bacterium HMT 922
081BB142 Peptostreptococcaceae [XI][G−7] bacterium HMT 081
Catonella
[Eubacterium]_brachy
Adj. p=0.043
[Eubacterium]_yurii_group
97da5e92edb74c87d1a5b4ea0d3e563e
Adj. p=0.026
