# Supplementary material for: Suspension of oral hygiene practices highlights key bacterial shifts in saliva, tongue, and tooth plaque during gingival inflammation and resolution
Source: ISME Commun. 2023 Mar 25;3:23. doi: 10.1038/s43705-023-00229-5 (PMC10039884; doi:10.1038/s43705-023-00229-5)
Supplement: Supplementary file 18 — Figure S18 [file 43705_2023_229_MOESM18_ESM.pdf]

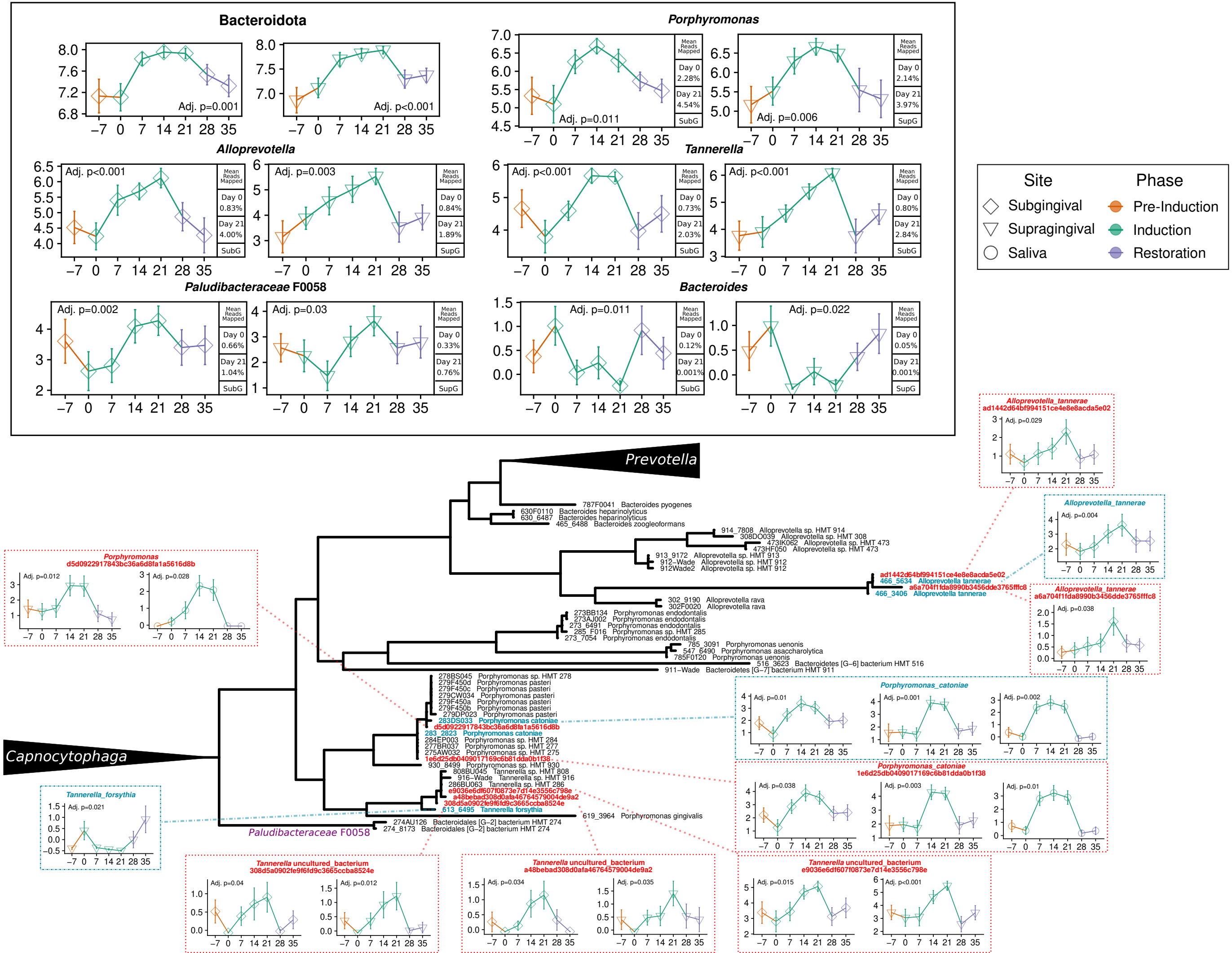

Bacteroidota
Adj. p=0.001
Adj. p<0.001
Porphyromonas
Adj. p=0.011
Adj. p=0.006
Alloprevotella
Adj. p<0.001
Adj. p=0.003
Tannerella
Adj. p<0.001
Adj. p<0.001
Paludibacteraceae F0058
Adj. p=0.002
Adj. p=0.03
Bacteroides
Adj. p=0.011
Adj. p=0.022
Mean Reads Mapped
Day 0 2.28% Day 21 4.54% SubG
Day 0 2.14% Day 21 3.97% SupG
Day 0 0.83% Day 21 4.00% SubG
Day 0 0.84% Day 21 1.89% SupG
Day 0 0.73% Day 21 2.03% SubG
Day 0 0.80% Day 21 2.84% SubG
Day 0 0.66% Day 21 1.04% SubG
Day 0 0.33% Day 21 0.76% SupG
Day 0 0.12% Day 21 0.001% SubG
Day 0 0.05% Day 21 0.001% SupG
Site
Subgingival
Supragingival
Saliva
Phase
Pre-Induction
Induction
Restoration
Prevotella
Capnocytophaga
Paludibacteraceae F0058
787F0041 Bacteroides pyogenes
630F0110 Bacteroides heparinolyticus
630_6487 Bacteroides heparinolyticus
465_6488 Bacteroides zoogleoformans
914_7808 Alloprevotella sp. HMT 914
308DO039 Alloprevotella sp. HMT 308
473IK062 Alloprevotella sp. HMT 473
473HF050 Alloprevotella sp. HMT 473
913_9172 Alloprevotella sp. HMT 913
912–Wade Alloprevotella sp. HMT 912
912Wade2 Alloprevotella sp. HMT 912
ad1442d64bf994151ce4e8e8acda5e02
466_5634 Alloprevotella tannerae
a6a704f1fda8990b3456dde3765fffc8
466_3406 Alloprevotella tannerae
302_9190 Alloprevotella rava
302F0020 Alloprevotella rava
273BB134 Porphyromonas endodontalis
273AJ002 Porphyromonas endodontalis
273_6491 Porphyromonas endodontalis
285_F016 Porphyromonas sp. HMT 285
273_7054 Porphyromonas endodontalis
785_3091 Porphyromonas uenonis
547_6490 Porphyromonas asaccharolytica
785F0120 Porphyromonas uenonis
516_3623 Bacteroidetes [G–6] bacterium HMT 516
911–Wade Bacteroidetes [G–7] bacterium HMT 911
278BS045 Porphyromonas sp. HMT 278
279F450d Porphyromonas pasteri
279F450c Porphyromonas pasteri
279CW034 Porphyromonas pasteri
279F450a Porphyromonas pasteri
279F450b Porphyromonas pasteri
279DP023 Porphyromonas pasteri
283DS033 Porphyromonas catoniae
d5d0922917843bc36a6d8fa1a5616d8b
283_2823 Porphyromonas catoniae
284EP003 Porphyromonas sp. HMT 284
277BR037 Porphyromonas sp. HMT 277
275AW032 Porphyromonas sp. HMT 275
1e6d25db0409017169c6b81dda0b1f38
930_8499 Porphyromonas sp. HMT 930
808BU045 Tannerella sp. HMT 808
916–Wade Tannerella sp. HMT 916
286BU063 Tannerella sp. HMT 286
e9036e6df607f0873e7d14e3556c798e
a48bebad308d0afa46764579004de9a2
308d5a0902fe9f6fd9c3665ccba8524e
613_6495 Tannerella forsythia
619_3964 Porphyromonas gingivalis
274AU126 Bacteroidales [G–2] bacterium HMT 274
274_8173 Bacteroidales [G–2] bacterium HMT 274
Alloprevotella_tannerae
ad1442d64bf994151ce4e8e8acda5e02
Adj. p=0.029
Alloprevotella_tannerae
Adj. p=0.004
Alloprevotella_tannerae
a6a704f1fda8990b3456dde3765fffc8
Adj. p=0.038
Porphyromonas
d5d0922917843bc36a6d8fa1a5616d8b
Adj. p=0.012
Adj. p=0.028
Porphyromonas_catoniae
Adj. p=0.01
Adj. p=0.001
Adj. p=0.002
Porphyromonas_catoniae
1e6d25db0409017169c6b81dda0b1f38
Adj. p=0.038
Adj. p=0.003
Adj. p=0.01
Tannerella_forsythia
Adj. p=0.021
Tannerella uncultured_bacterium
308d5a0902fe9f6fd9c3665ccba8524e
Adj. p=0.04
Adj. p=0.012
Tannerella uncultured_bacterium
a48bebad308d0afa46764579004de9a2
Adj. p=0.034
Adj. p=0.035
Tannerella uncultured_bacterium
e9036e6df607f0873e7d14e3556c798e
Adj. p=0.015
Adj. p<0.001
